# Trafficking through the blood–brain barrier is directed by core and outer surface components of layer‐by‐layer nanoparticles

**DOI:** 10.1002/btm2.10636

**Published:** 2023-12-28

**Authors:** Nicholas G. Lamson, Andrew J. Pickering, Jeffrey Wyckoff, Priya Ganesh, Elizabeth A. Calle, Joelle P. Straehla, Paula T. Hammond

**Affiliations:** ^1^ Koch Institute for Integrative Cancer Research, Massachusetts Institute of Technology Cambridge Massachusetts USA; ^2^ Department of Chemical Engineering Massachusetts Institute of Technology Cambridge Massachusetts USA; ^3^ Department of Materials Science and Engineering Massachusetts Institute of Technology Cambridge Massachusetts USA; ^4^ Department of Surgery Massachusetts General Hospital Boston Massachusetts USA; ^5^ Department of Pediatric Oncology Dana‐Farber Cancer Institute Boston Massachusetts USA; ^6^ Division of Pediatric Hematology/Oncology Boston Children's Hospital Boston Massachusetts USA; ^7^ Broad Institute of MIT and Harvard Cambridge Massachusetts USA; ^8^ Institute for Soldier Nanotechnologies, Massachusetts Institute of Technology Cambridge Massachusetts USA

**Keywords:** blood–brain barrier, drug delivery, in vitro models, intracellular trafficking, intravital imaging, layer‐by‐layer, nanoparticles

## Abstract

Drug‐carrying nanoparticles are a promising strategy to deliver therapeutics into the brain, but their translation requires better characterization of interactions between nanomaterials and endothelial cells of the blood–brain barrier (BBB). Here, we use a library of 18 layer‐by‐layer electrostatically assembled nanoparticles (NPs) to independently assess the impact of NP core and surface materials on in vitro uptake, transport, and intracellular trafficking in brain endothelial cells. We demonstrate that NP core stiffness determines the magnitude of transport, while surface chemistry directs intracellular trafficking. Finally, we demonstrate that these factors similarly dictate in vivo BBB transport using intravital imaging through cranial windows in mice. We identify that hyaluronic acid surface chemistry increases transport across the BBB in vivo, and flow conditions are necessary to replicate this finding in vitro. Taken together, these findings highlight the importance of assay geometry, cell biology, and fluid flow in developing nanocarriers for delivery to the brain.

**Translational Impact Statement**

Nanoscale drug carriers hold great potential for improving delivery of therapeutics across the blood–brain barrier (BBB) to treat brain tumors or other neurological diseases, and this work uses the modular layer‐by‐layer system of polymeric nanoparticle assembly to independently define how material stiffness and surface chemistry drive particle behavior at the BBB, allowing for more rational design and therefore more rapid clinical translation of brain‐targeted drug carriers.

## 1 INTRODUCTION

Neurological disorders, including but not limited to brain tumors and neurological diseases, are the leading global cause of years of life lost and second leading cause of death, according to data published in 2019.^1^ However, development of new treatments for neurologic disorders has been challenging, largely due to poor drug transport across the blood–brain barrier (BBB)—the specialized vascular lining of the central nervous system.^2^ At least 95% of newly discovered candidate therapies are excluded from entering the brain^3^—even in glioma tumors and neurological diseases commonly associated with “leaky” BBBs^4^, ^5^—and there is a critical need to develop drug carriers that can transport therapeutic cargoes across the BBB.

**FIGURE 1:**
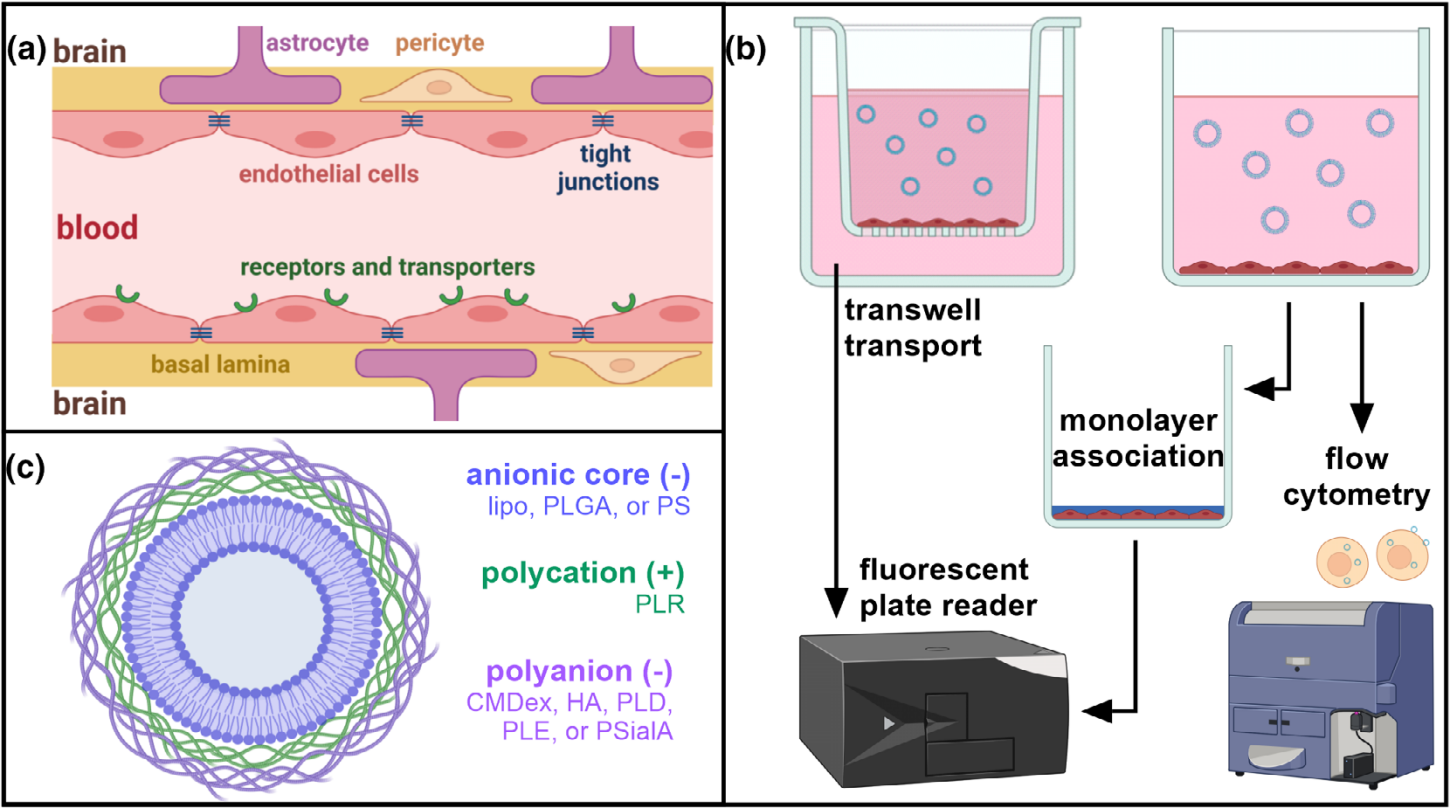
Probing nanomaterial uptake behavior in the blood–brain barrier (BBB) using fluorescently labeled layer‐by‐layer nanoparticles (LbL‐NPs). (a) The BBB derives its barrier properties primarily from endothelial cells, which are stitched together with tight junction proteins, but express receptors, transporters, and other machinery for cell‐mediated uptake. (b) Using brain endothelial cell lines, three common assays for nanomaterial uptake for in vitro BBB models are transwell transport, monolayer association, and flow cytometry using fluorescently tagged nanoparticles. The output for transwell transport is fluorescence of material that has passed through the cell layer at a given time, while monolayer association and flow cytometry respectively report fluorescence of cell populations or individual cells. (c) LbL‐NPs—used here to cross‐compare the effects of nanoparticle stiffness and surface chemistry—comprise one of three fluorescently labeled and negatively charged cores: anionic liposomes (lipo), acid‐ terminated poly (lactic‐*co*‐glycolic acid) (PLGA), or carboxylated polystyrene (PS). The cores are electrostatically layered with a polycation layer, in this case poly‐l‐arginine (PLR). Finally, a polyanion outer surface layer is added. The outer layers used here are carboxymethyldextran (CMDex), hyaluronic acid (HA), poly‐l‐aspartic acid (PLD), poly‐l‐glutamic acid (PLE), and polysialic acid (PSialA), giving an overall negative charge to the NP surface.

Capillaries, the smallest blood vessels in the brain, are lined with specialized endothelial cells surrounded by a basal lamina composed of extra by BBB‐specific extracellular matrix, and are supported by pericytes and astrocytes (Figure 1a).^6^ These endothelial cells comprise the majority of the transport barrier, with tight junctions stitching the cells together to exclude passive and non‐specific transport between the cells, including most small molecules; uptake of nutrients, therapeutics, and nanocarriers relies on cell‐mediated, active transport to cross the BBB.^6^ For this reason, the early stages of brain therapeutic development rely on in vitro BBB models that recapitulate endothelial cell morphology and transport mechanisms to screen candidates for brain delivery, necessitating standardized, high‐throughput in vitro models to develop new delivery vehicles.^7^

Within the field of in vitro BBB modeling, there are several available types of endothelial cell, each of which presents its own benefits and drawbacks. Primary brain endothelial cells, isolated from patient samples, are the most biologically sophisticated, but lose their brain‐specific endothelial phenotypes quickly in cell culture.^8^ Endothelial cells derived from induced pluripotent stem cells are biologically diverse,^9^ but their ability to recapitulate a brain endothelial phenotype remains controversial.^10^ Finally, immortalized cell lines are less genetically diverse, but have stable expression of canonical BBB genes encoding junctional and transport proteins.^11^, ^12^ For this reason, we used the human‐derived hCMEC/D3 immortalized microcapillary endothelial cell line in this study. hCMEC/D3 has an endothelial phenotype, characteristic BBB protein expression,^13^, ^14^, ^15^, ^16^, ^17^ and recapitulates the endolysosomal and transcytosis systems of primary brain endothelial cells.^18^

Beyond the type of endothelial cell used, there are several model geometries—two‐ dimensional (2D) or three‐dimensional (3D)—and models with or without media flow available. Each combination of these factors offers its own advantages for inter‐lab consistency, ease of handling, and/or recapitulation of the physical environment of the brain capillaries.^19^ For example, there are several 3D microfluidic models currently under development for screening of therapeutics or material libraries.^20^, ^21^, ^22^ However, these remain difficult to standardize across labs or to scale for high‐throughput studies. For these reasons, we chose to focus here on three broadly accessible, 2D static model assays to study nanoparticle (NP) uptake and transport in hCMEC/D3 BBB endothelial cells.

Despite ample literature describing small molecule and biologic transport in BBB models,^23^, ^24^ there has been limited study of and little standardization in methodology for assessing BBB transport of nanomaterials.^20^, ^25^ Three commonly used assays include NP association via flow cytometry,^26^, ^27^ monolayer association of NPs,^25^, ^28^ and transport of NPs across transwell‐grown cell monolayers^26^, ^28^ (Figure 1b). Within the transwell model, there is no consensus for how transwell pore size or monolayer development time affect transport properties and gene expression.^14^, ^16^, ^28^ By comparing how these geometrical parameters, along with resulting differences in barrier properties and gene expression, dictate hCMEC/D3 cell processing of a variety of NPs, we hypothesize that we can inform best practices for future, high‐throughput nanomaterials screens to speed development of new strategies for drug delivery to the brain, as well as identify current top candidates for drug nanocarriers that cross the BBB.

To complete this screen, we employ a curated library of layer‐by‐layer assembled NPs (LbL‐NPs) to examine how these BBB models extend to nanomaterial uptake and transport behavior at the BBB. Our LbL‐NP library combines three negatively charged NP cores—liposomes, poly(lactic‐*co*‐glycolic acid) (PLGA), and polystyrene (PS)—alone or with one of five polyanion outer layers—carboxymethyldextran (CMDex), hyaluronic acid (HA), poly‐l‐aspartic acid (PLD), poly‐l‐glutamic acid (PLE), or polysialic acid (PSialA)—to yield 18 formulations with defined core stiffness and surface chemistries (Figure 1c). Screening these NPs using the three previously described in vitro assays allows us to investigate how particle characteristics act as controlling factors for BBB uptake and transport, as well as how surface chemistry in particular dictates NP trafficking within hCMEC/D3 cells. Finally, to match the standard preclinical pipeline for therapeutic development, we use a select set of LbL‐NPs to compare our in vitro BBB models to in vivo BBB transport in mice. We apply intravital multiphoton imaging through cranial windows to visualize NP concentrations in blood vessels and brain parenchyma, enabling us to calculate the NP permeability across the BBB.^22^, ^29^ Comparing results across in vitro and in vivo assays highlights promises and pitfalls of each in vitro measure and informs design of nanocarriers for drug delivery to the brain.

## 2 RESULTS AND DISCUSSION

### 2.1 Optimization of hCMEC/D3 BBB transwell model

**FIGURE 2:**
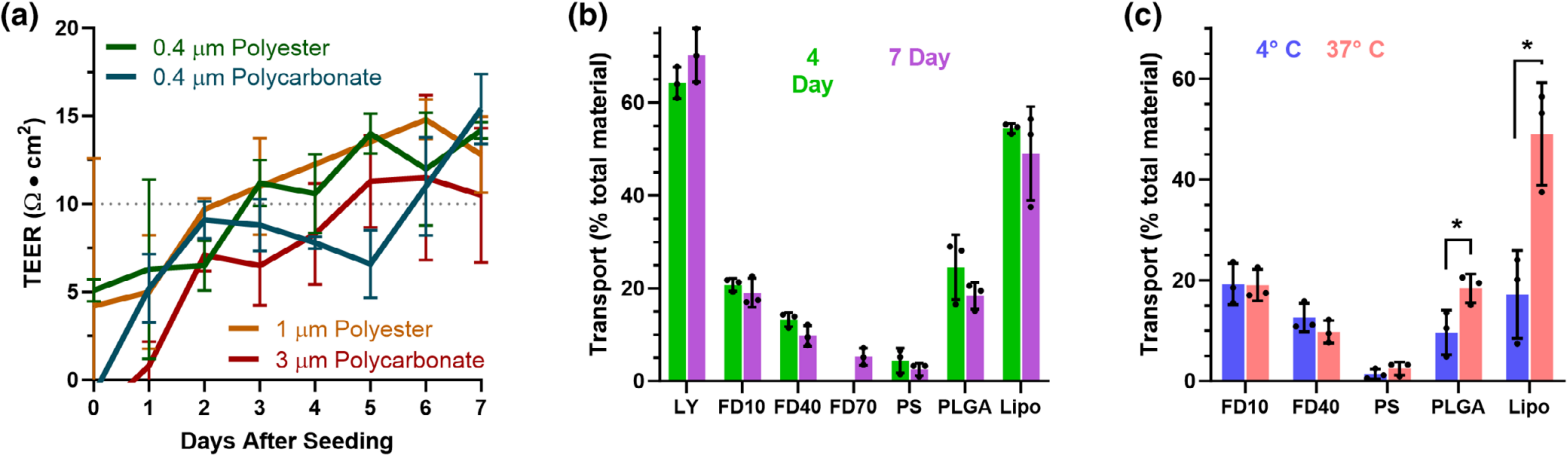
hCMEC/D3 cells grown on transwell inserts with 1 μm pores produce monolayers with proper barrier integrity to examine active transport of nanomaterials. (a) Across three pore sizes and two transwell membrane materials, transendothelial electrical resistance (TEER) of hCMEC/D3 monolayers increased, then plateaued at 3–5 days post seeding. (b) On 1 μm pore polyester membranes, transport of paracellular permeability markers lucifer yellow (LY), 10 kDa FITC‐dextran (FD10), 40 kDa FITC Dextran (FD40), and nanoparticle cores did not differ between monolayers grown for 4 or 7 days. FD70 was examined on 7‐day monolayers only. (c) In 7‐day monolayers, performing transport studies at 4°C did not substantially diminish passive transport of diffusion markers FD10 and FD40. In contrast, poly(lactic‐*co*‐glycolic acid) (PLGA) nanoparticle and liposome transport slowed substantially, indicating that their uptake across monolayers is a predominantly active, transcellular process. Data display mean ± standard deviation for *n* = 3 transwell insert replicates (for TEER) or *n* = 3 biological plate replicates (for material transport). *p < 0.05 by Mann–Whitney *U* test for nonparametric data.

We began by characterizing the impact of filter choice and experiment duration on hCMEC/D3 endothelial cell monolayers on transwell membranes, to understand how these parameters affect the transport model. First, we seeded cells onto four different types of transwell filters—0.4 μm pore polyester or polycarbonate, 1 μm pore polyester, or 3 μm pore polycarbonate—all coated with type 1 collagen to provide a suitable attachment and growing surface for the cells.^13^ As indicated by trans‐endothelial electrical resistance (TEER), a surrogate measure for barrier function of the monolayers,^30^ cells on polyester substrates formed a stable barrier—TEER ≥10 Ω cm^2^ ^17^—within 4 days (Figure 2a). This is consistent with other published studies,^12^ as is a more gradual increase in barrier strength over at least 4–7 days.^16^, ^31^ While all four filter types yielded similar TEER values, media leakage through the 3 μm pore polycarbonate during handling complicated experimental procedures. Given this, and our concern that the smallest (0.4 μm) pores may provide non‐negligible diffusive resistance to 100–200 nm nanomaterials, we used 1 μm pore polyester transwell filters for further investigation.

We next investigated barrier behavior of transwell monolayers at 4 or 7 days past cell seeding, using passive diffusion markers to assess permeability through tight junctions between cells. As expected, smaller markers—0.44 kDa Lucifer Yellow (LY) and 10 kDa FITC‐Dextran (FD10)—are more permeable than larger substrates—40 kDa FITC‐Dextran (FD40) and 70 kDa FITC‐Dextran (FD70) after a 24‐h incubation period (Figure 2b).^11^ There were no significant differences in permeability between 4‐and 7‐day monolayers, indicating that model development time does not substantially affect passive diffusion after 4 days. However, rapid passage of LY in particular corroborates previous reports that hCMEC/D3 monolayers do not recapitulate proper BBB exclusion of hydrophilic small molecules, but do provide a proper barrier to macromolecules and nanomaterials.^32^ For anionic, 100 nm diameter PS, PLGA, and liposomal NPs, there were no significant transport differences based on monolayer seeding time. Notably, PLGA and liposomal NPs exhibited substantially higher transport than fluorescent dextrans, despite the fact that dextrans would encounter less diffusive resistance due to their smaller sizes. This indicates that the nanomaterials cross the monolayers via an active process, rather than passively diffusing between the cells. To validate our hypothesis that PLGA and liposome transport is driven by cell‐mediated, energy‐dependent processes, we compared seven‐day monolayers at physiologic temperature to monolayers that were chilled to 4°C immediately after treatment addition (Figure 2c). While passive diffusion was unchanged, PLGA NPs and liposomes crossed the cell monolayers at significantly lower rates at 4°C, confirming the transport to be active. It is noted that the transition temperature for each of the individual phospholipids in the liposome formulation is above 50°C,^33^ so we do not expect that changes in NP stiffness between 37 and 4°C contribute to this difference in transport.

### 2.2 Biological characterization of hCMEC/D3 models

**FIGURE 3:**
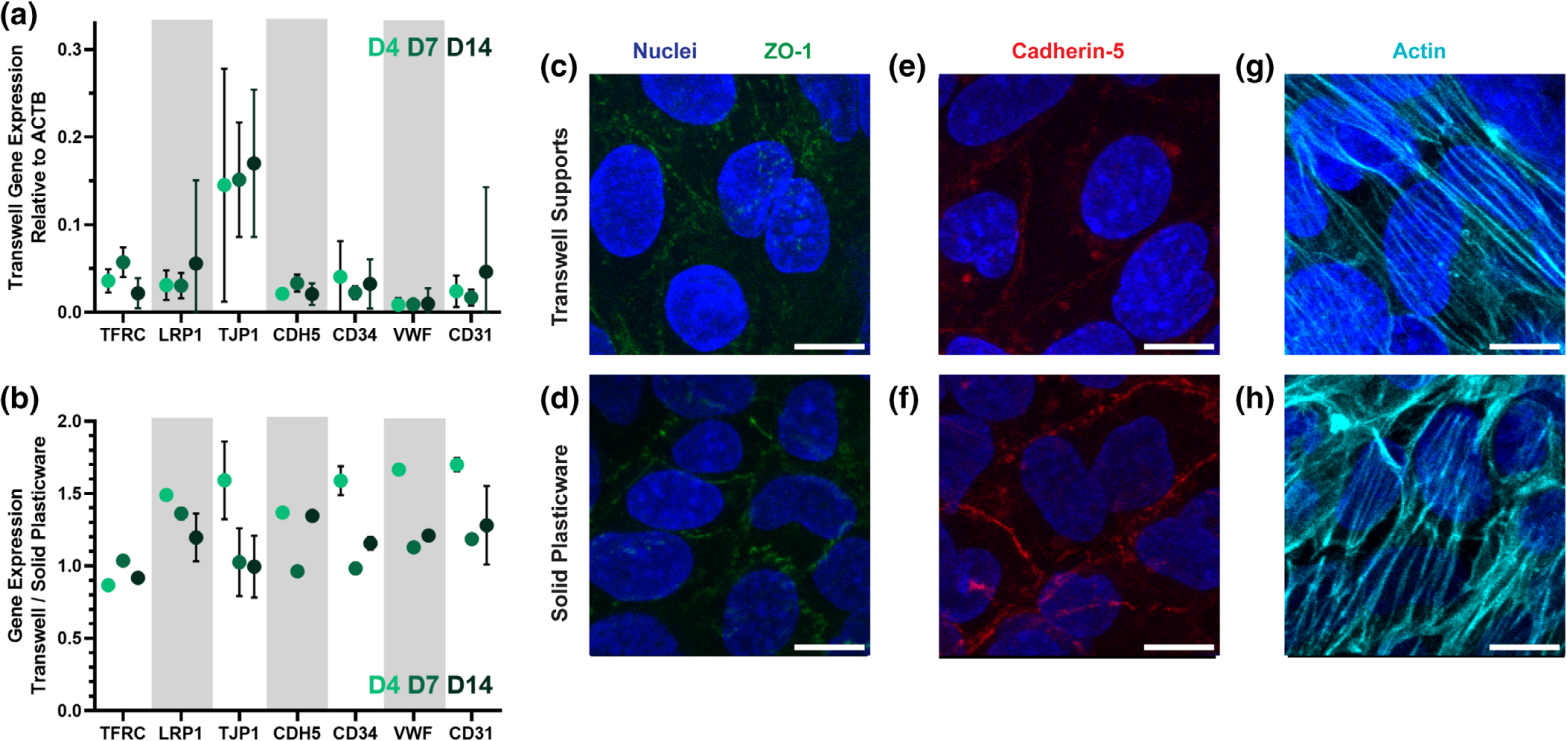
hCMEC/D3 cells demonstrate consistent gene expression and localization of key cellular proteins across experimental conditions. (a) Gene expression via by RT‐quantitative polymerase chain reaction (qPCR) is similar over time, comparing cells grown on transwell filters for 4, 7, or 14 days. For each gene, there was no significant difference between groups by Kruskal–Wallis *H* test, and error bars display standard deviation of three biological replicates at passage numbers 2, 6, and 10. (b) Likewise, the difference in gene expression did not vary substantially between cell monolayers grown on transwell filters and those grown on solid plasticware. (c) By confocal imaging, ZO‐1 in both transwell‐grown cells and (d) plasticware‐grown cells show discontinuous rings of tight junctions around each cell. (d) The same pattern is seen in cadherin‐5 in both transwell‐grown and (f) plasticware‐grown cells. (g) Actin staining in transwell‐grown and (h) plasticware‐grown cells shows cooperative filament organization across several cells. Scale bars display 10 μm.

Having validated barrier properties of transwell‐grown hCMEC/D3 monolayers, we next investigated expression of endothelial genes and canonical BBB transporters to best compare to published works that have previously characterized these cells. By preparation of complimentary DNA (cDNA) libraries and subsequent analysis via quantitative polymerase chain reaction (qPCR), we found no significant differences in seven genes—transferrin receptor (TFRC),^11^ low density lipoprotein‐related receptor 1 (LRP1),^14^ zonula occludens 1 (ZO‐1 or TJP1),^8^ VE‐cadherin (CDH5),^12^ CD34,^34^ Von Willebrand Factor (VWF),^32^ and PECAM‐1 (CD31)^32^—across hCMEC/D3 monolayers developed for 4, 7, or 14 days (Figure 3a). Relative transcript levels matched closely with in vitro BBB studies across the literature,^16^, ^35^ and are tabulated in Supplementary Table 1. To extend these findings, we examined gene expression in cell monolayers grown on collagen‐coated, non‐porous plasticware for the same durations of time (Figure 3b). For the TFRC, expression in transwell‐grown and plasticware‐grown cells was not significantly different. For the remaining genes, however, there was significantly higher expression of the classical endothelial markers in transwell‐grown cells on day 4, compared to solid plastic tissue culture, while expression levels between the two systems more closely match—that is, a gene expression ratio closest to 1—at days 7 and 14. Therefore, to draw the most meaningful comparisons between the two assay formats, we decided to move forward with seven‐day monolayers for the remainder of our experiments. By immunofluorescence confocal imaging, we confirmed that monolayers grown in both systems were structurally similar, with expected patterns of localization for ZO‐1 (Figure 3c,d), Cadherin‐5 (Figure 3e,f), and coordinated F‐actin fibers across multiple cells (Figure 3g,h). The discontinuous nature of ZO‐1 and cadherin 5 around each cell, in particular, is consistent with published literature and supports our findings that hCMEC/D3 monolayers are leaky to small molecules, but can provide sufficient transport barriers for macromolecules and nanomaterials.^12^, ^13^, ^32^, ^36^

### 2.3 Combinatorial LbL‐NP library

**FIGURE 4:**
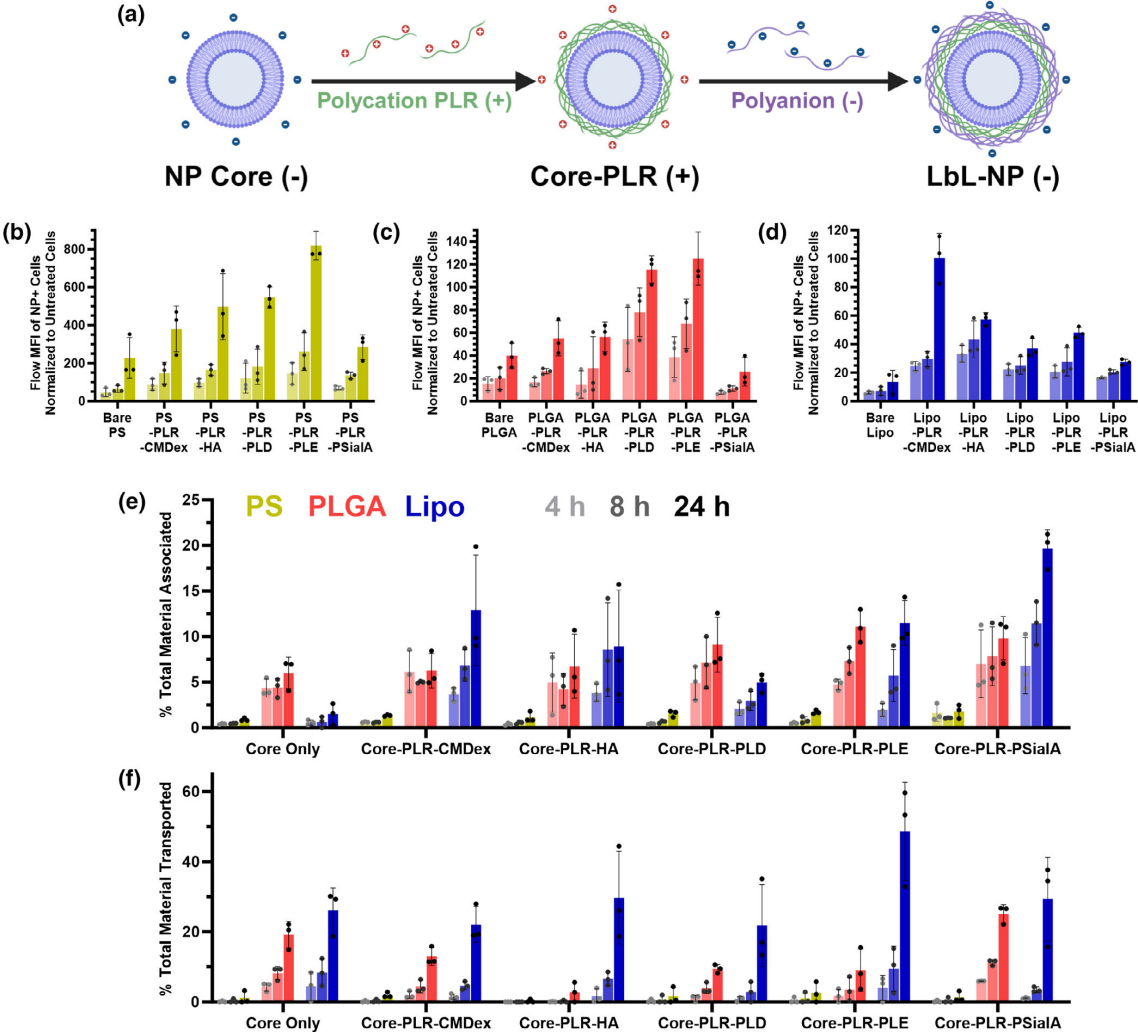
hCMEC/D3‐based models of the blood–brain barrier (BBB) differ in their uptake of a library of layer‐by‐layer assembled nanoparticles. (a) Layer‐by‐layer electrostatically assembled nanoparticles (LbL‐NPs) are formulated by incubating charged NP cores with oppositely charged polymer solutions, followed by removal of non‐adsorbed polymer. For this work, anionic cores, one polycation (PLR) and five polyanions were employed to create polymer bilayers. (b) In cells grown on solid plasticware and treated with PS core nanoparticles, flow cytometry median fluorescence intensity measured at 4, 8 and 24 h for nanoparticle positive cells differed by outer layer chemistry, with PLE outperforming the others. This trend was mirrored closely in (c) cells treated with poly(lactic‐*co*‐glycolic acid) (PLGA) core nanoparticles, while (d) cells treated with liposome‐core nanoparticles showed greatest uptake with CMDex capped particles instead. (e) Monolayer association in cells grown on solid plasticware indicated a preferred uptake based on NP cores with medium or low stiffness, with the exception of bare liposomes. (f) Likewise, in monolayers grown on transwells, soft liposome‐core nanoparticles transported across the monolayer to a greater degree than the stiffer polymer nanoparticles, regardless of surface chemistry. Data are reported as arithmetic mean ± standard deviation of three plate replicates, with three technical replicate wells per treatment per plate.

We next created a library of LbL‐NPs to compare in cell uptake and association assays (Figure 4a). The modularity of LbL‐NPs allows for combinatorial screening of NP core materials and surface chemistries to assess the independent effects of changing each factor on their biological activity.^37^ For our library, anionic (phospholipid) liposomes, acid‐terminated PLGA NPs, or carboxylated PS NPs with diameters of 80–100 nm and covalently bound fluorophores were simply mixed with poly‐l‐arginine (PLR) in HEPES buffer; following removal of non‐adsorbed polymer by tangential flow filtration (TFF), successful PLR adsorption was confirmed by zeta potential charge conversion to >40 mV. By the same process, the PLR‐coated NPs were then layered with one of five polyanions—CMDex, HA, PLD, PLE, or PSialA—at ratios chosen to give anionic outer surfaces with zeta potential more negative than −30 mV; these polyions cover a range of synthetic polypeptides, synthetic carbohydrates, and naturally occurring carbohydrates. Characterization by dynamic light scattering (DLS) is displayed in Table 1. Cryo‐transmission electron microscopy (TEM) images of select formulations can be found in Supplementary Figure 1, and previous work from our lab has demonstrated via scanning electron microscopy (SEM) and atomic force microscopy (AFM) that this process produces uniform, spherical NPs.^38^, ^39^ Because cationic particles undergo non‐specific cellular interactions and rapid clearance from circulation in vivo,^40^ only the anionic NP cores and LbL‐NPs with polyanion outer layers advanced into biological assays.

**TABLE 1:** NP characteristics measured by dynamic light scattering. All values are displayed as arithmetic mean ± standard deviation of three measurements.

NP name	Number average size (nm)	Z‐average size (nm)	PDI	Zeta potential (mV)	Zeta deviation (mV)
Bare liposome	82.9 ± 5.9	105.2 ± 1.5	0.11 ± 0.03	−52.2 ± 0.9	14.8 ± 0.6
Lipo‐PLR	113.9 ± 3.7	141.7 ± 3.3	0.08 ± 0.05	45.0 ± 0.4	10.3 ± 3.9
Lipo‐PLR‐CMDex	102.1 ± 13.8	167.8 ± 4.4	0.23 ± 0.09	−34.8 ± 1.0	9.0 ± 3.5
Lipo‐PLR‐HA	88.2 ± 7.5	124.9 ± 2.1	0.20 ± 0.01	−33.0 ± 1.1	6.2 ± 1.1
Lipo‐PLR‐PLD	81.6 ± 5.6	131.9 ± 4.7	0.22 ± 0.02	−42.9 ± 7.2	10.5 ± 2.5
Lipo‐PLR‐PLE	82.6 ± 8.5	130.2 ± 2.6	0.21 ± 0.01	−49.0 ± 3.8	35.7 ± 9.8
Lipo‐PLR‐PSialA	104.2 ± 3.1	144.6 ± 3.4	0.11 ± 0.03	−50.0 ± 2.7	23.0 ± 14.0
Bare PLGA	85.0 ± 4.9	136.7 ± 3.1	0.17 ± 0.02	−51.0 ± 2.3	16.9 ± 1.4
PLGA‐PLR	91.8 ± 5.7	144.8 ± 4.3	0.18 ± 0.01	42.0 ± 2.2	12.5 ± 7.0
PLGA‐PLR‐CMDex	85.0 ± 4.9	167.8 ± 4.4	0.17 ± 0.02	−51.0 ± 2.3	5.7 ± 1.3
PLGA‐PLR‐HA	88.5 ± 3.8	144.3 ± 3.9	0.25 ± 0.05	−30.5 ± 0.3	4.8 ± 1.1
PLGA‐PLR‐PLD	75.8 ± 8.7	148.9 ± 5.5	0.30 ± 0.05	−41.8 ± 1.3	5.4 ± 1.5
PLGA‐PLR‐PLE	72.7 ± 10.8	215.9 ± 9.0	0.17 ± 0.04	−42.6 ± 0.5	4.4 ± 2.1
PLGA‐PLR‐PSialA	78.7 ± 10.2	152.2 ± 1.9	0.36 ± 0.04	−43.5 ± 3.7	5.7 ± 1.3
Bare PS	107.7 ± 7.0	131.4 ± 2.3	0.07 ± 0.05	−59.0 ± 0.6	6.3 ± 0.6
PS‐PLR	105.2 ± 2.0	132.4 ± 2.4	0.07 ± 0.02	60.3 ± 3.4	8.8 ± 2.4
PS‐PLR‐CMDex	148.5 ± 11.1	143.2 ± 1.1	0.11 ± 0.08	−52.5 ± 1.8	8.0 ± 0.3
PS‐PLR‐HA	98.7 ± 6.7	120.8 ± 24.5	0.18 ± 0.05	−31.9 ± 1.0	5.6 ± 1.1
PS‐PLR‐PLD	108.6 ± 7.4	143.1 ± 4.5	0.08 ± 0.05	−43.7 ± 0.6	18.4 ± 4.1
PS‐PLR‐PLE	99.4 ± 10.2	166.0 ± 3.7	0.28 ± 0.04	−46.7 ± 2.3	8.9 ± 0.9
PS‐PLR‐PSialA	109.2 ± 2.4	133.2 ± 1.1	0.01 ± 0.00	−54.0 ± 3.3	13.3 ± 2.1

Abbreviations: CMDex, carboxymethyldextran; HA, hyaluronic acid; PDI, polydispersity index; PLGA, poly(lactic‐*co*‐glycolic acid); PLD, poly‐l‐aspartic acid; PLE, poly‐l‐glutamic acid; PSialA, polysialic acid; PS, polystyrene.

### 2.4 Validation of hCMEC/D3 BBB models using LbL‐NPs

We applied our LbL‐NP library to the three orthogonal uptake or transport assays using the hCMEC/D3 endothelial cells: flow cytometry and monolayer association using cells grown in standard plasticware, and transport across a transwell monolayer. For flow cytometry, treated cells are rinsed to remove any NPs that have not been taken up or bound to the cell surface, and the cells are dissociated for cell counting and fluorescence analysis. Differing fluorophores and/or brightness between the NP cores precludes direct comparison across the groups. However, within their formulation groups, PS core LbL‐NPs (Figure 4b), PLGA‐core LbL‐NPs (Figure 4c), and liposome core LbL‐NPs (Figure 4d) generally displayed increases in median fluorescence intensity over the bare NP cores, indicating that LbL functionalization generally increases interactions with the hCMEC/D3 cells; for the purposes of this discussion, these interactions or associations of NPs for cells will be referred to as NP uptake, and includes NPs internalized or bound to the cell membrane surface. For PS or PLGA core LbL‐NPs, the polypeptide outer layers PLD and PLE conferred an uptake advantage, while liposome core LbL‐NPs with polysaccharide outer layers CMDex and HA showed greater uptake than other formulations. However, most of the groups showed only up to twofold to threefold difference between LbL‐NP formulations, which is consistent with other flow cytometry studies of NP surface chemistry impacts on uptake in hCMEC/D3,^26^ as well as in other cell and tissue types.^41^, ^42^ Surprisingly, despite previous reports that sialic acid functionalization of NP surfaces improves BBB transport,^43^ the PSialA coated LbL‐NPs—especially those with PLGA cores—did not undergo improved uptake in this assay.

We next examined the LbL‐NP library for uptake using monolayer association (Figure 4e)—in monolayer association, treated cells are rinsed to remove any NPs that have not been internalized or bound to cell surfaces, then the cells are homogenized using a combination of dimethyl sulfoxide (DMSO) and heparin sulfate before examining for fluorescent NP content using a plate reader—and for transport through monolayers grown on transwell supports (Figure 4f). Because both assays enable creation of NP fluorescence calibration curves, both allow for direct cross‐comparison between LbL‐NP formulations with different NP cores. In both cases, uptake or transport of the compliant, liposome‐based LbL‐NPs was greater than or similar to the stiffer polymeric core NPs with matched outer surface layers. Previous work from our lab has shown these LbL‐NPs resist disassembly and retain the majority of their polymeric coatings through 24 h in cell culture conditions.^44^ Thus we hypothesize that differences can be primarily attributed to the stiffness and deformability of the core material; we have demonstrated previously that the elastic modulus (*E*) of these liposomal particles is approximately 5–10 kPa,^45^ while bulk elastic moduli for semicrystalline PLGA and glassy PS have been reported at approximately 600 MPa and 3.7 GPa, respectively.^46^, ^47^ This improved performance of softer nanomaterial transport is consistent with studies of nanogels in static hCMEC/D3 systems,^48^ while limited studies in systems with fluid flow have not yet reached consensus on the relationship between particle stiffness and BBB transport.^20^, ^22^, ^49^ By comparing surface chemistries with the same core material, we again observed that LbL functionalization increases monolayer association (Figure 4d) for most formulations over the bare core, though the differences were less stark than those observed by flow cytometry. In contrast, we did not observe this relationship for transwell transport (Figure 4e), in which bare PS and PLGA particles transported as well as, or better than, several of the polymer functionalized formulations and there were not statistical enhancements for the liposomal formulations with LbL surfaces.

While measuring NP fluorescence in monolayer association and transwell transport assays, we found that it was necessary to fully homogenize and solubilize our samples to obtain robust quantitative data. In the transwell assay, reading fluorescence directly from the lower chamber implied 160–180% of theoretically maximum NP transport for PLE‐coated liposomes (Supplementary Figure 2a). By homogenizing the samples—including samples used for the calibration curves—with DMSO to dissolve the liposomes along with addition of heparin sulfate to sequester PLR away from the lipid‐conjugated, sulfo‐Cy5 fluorophore, we obtained the data displayed in Figure 4d,e. Because homogenizing the particles led to a brighter but more consistent fluorescence (Supplementary Figure 2b), we concluded that the Cy5 fluorophore attached to the liposome core is partially self‐quenching, and liposome breakup allows for higher activity of solubilized lipid‐fluorophore conjugates. By contrast, PLGA core LbL‐NPs, which do not self‐quench, do not demonstrate such discrepancies (Supplementary Figure 2c). The apparent dequenching of the Cy5 in some the liposomal LbL‐NPs thus implied that the fluorophore‐linked lipids forming the NP core were dissociating from one another as they transported across the cell monolayers. It is well characterized in endothelial cells that nanomaterials are sorted post‐internalization into a variety of transport or processing pathways; of these the two most common are transcytosis—shuttling across the cells in vesicles with relatively unchanging, physiological conditions—and endolysosomal processing—packaging into vesicles with increasingly hostile enzymatic conditions and acidic pH designed to degrade their contents.^50^ Based on these behaviors, and the apparent degradation of some of our liposomal LbL‐NP formulations, we hypothesized that while core identity has the largest impact on the total amount of NP taken up and transported, NP surface chemistry dictates sorting into the intracellular pathways by which particles are processed through the cells.

**FIGURE 5:**
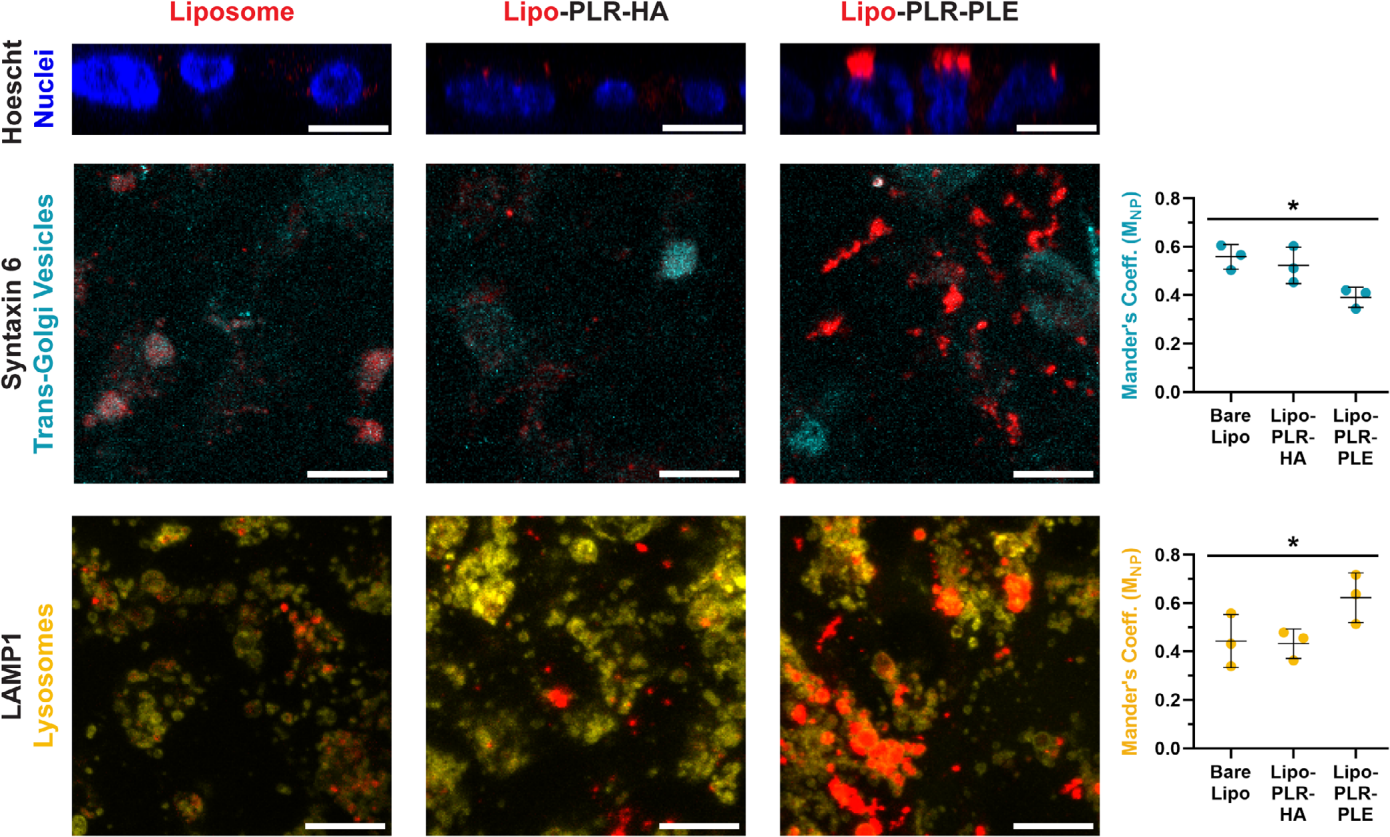
Outer layer chemistries on liposome‐core layer‐by‐layer electrostatically assembled nanoparticles (LbL‐NPs) influence cellular uptake and trafficking in hCMEC/D3 monolayers grown on transwell supports. In orthogonal projections, bare liposomes and HA‐coated LbL‐NPs (Lipo‐PLR‐HA) can be seen distributing throughout the thickness of the monolayers, while PLE‐coated NPs (Lipo‐PLR‐PLE) are concentrated in large aggregates near the apical surface. Bare liposomes and HA‐coated NPs colocalized most strongly with the Syntaxin 6 marker of transcytosis machinery, which was confirmed by their higher Mander's coefficients with this marker. LAMP1 staining was largely independent of NP signal for liposomes and HA‐coated NPs, but aggregated PLE‐coated NP signals appear to be enclosed in lysosomal vesicles. Colocalization analyses likewise indicated higher Mander's coefficients for PLE‐coated NP overlap with LAMP1. Scale bars display 10 μm. Data display averages of three technical replicate images on three replicate monolayers from different hCMEC/D3 cell populations. * Treatment groups are statistically different (*p* < 0.05) by Kruskal–Wallis *H* test.

To test this hypothesis, we selected three LbL‐NP formulations (Supplementary Table 2) with liposome cores to examine their intracellular trafficking by confocal microscopy in transwell‐grown monolayers: bare liposomes which we hypothesize have no degradation during transport, HA‐coated NPs as an LbL‐NP formulation that we hypothesize undergoes minimal degradation, and PLE‐coated NPs as a formulation that we hypothesize undergoes significant degradation. After 8 h of NP treatment, we imaged z‐stacks of the NP‐treated monolayers to construct orthogonal views, and we observed that Cy5 signal for bare liposomes and HA‐coated LbL‐NPs presented as small spots scattered throughout the cell bodies (Figure 5). By contrast, the PLE‐coated LbL‐NPs demonstrated heavy localization of large, bright areas of NP signal to the apical surfaces of the cells. We confirmed that PLE NPs remain colloidally stable in cell culture media over this time period as measured by DLS (Supplementary Figure 3), and therefore this morphology is not likely an artifact of particles sedimenting to the cell surface, but rather indicates that PLE NPs traffic through different intracellular mechanisms than the bare liposomes or HA‐coated LbL‐NPs in hCMEC/D3 endothelial cells.

To further examine intracellular trafficking, we co‐stained identically treated cells with markers for proteins associated with several uptake and transport mechanisms. Neither clathrin nor caveolin‐1 showed any substantial colocalization with any of the NP particle formulations (Supplementary Figure 4). Similarly, none of the formulations colocalized strongly with the early endosome marker EEA1, indicating that the NPs are processed quickly through early endosomes and into other intracellular compartments. Live cells imaged for co‐uptake of NPs alongside 70 kDa fluorescent dextran demonstrated the most substantial signal overlap suggesting that a nonspecific uptake mechanism, such as macropinocytosis, is the strongest driver of NP internalization.^51^ We then investigated two major downstream pathways, and determined that bare liposomes and HA‐coated LbL‐NPs show strongest localization with Syntaxin 6 (Figure 5), a marker for Golgi‐associated vesicles that drive transcytosis.^50^ It should be noted that the weak and diffuse staining for Syntaxin 6 required imaging settings that produced background speckles across all samples; while this may artificially increase the Mander's coefficients for the entire image set, we do not believe that it affects the relative reduction in colocalization of PLE particles with the marker, as compared to the other NP formulations. By contrast, the PLE‐coated LbL‐NPs accumulate predominantly in vesicles with high expression of LAMP1, a lysosomal marker that denotes vesicles with high degradation capacity.^52^ This difference in colocalization supports our hypothesis that the NP surface chemistry is a controlling factor in determining how the materials sort into intracellular trafficking pathways. Based on the increased association between PLE‐coated LbL‐NPs and the previously reported discrepancies in fluorescence‐based transport data (Supplementary Figure 2a), we can conclude that this formulation is sorted into lysosomes for degradation by the endothelial cells. By contrast, the bare liposomes and HA‐coated LbL‐NPs that primarily associate with transcytosis machinery would be expected to remain intact as they transport through the cells, as implied by the fluorescence readings in our monolayer transport studies.

### 2.5 Comparison of cell culture models to BBB peremability in mice

**FIGURE 6:**
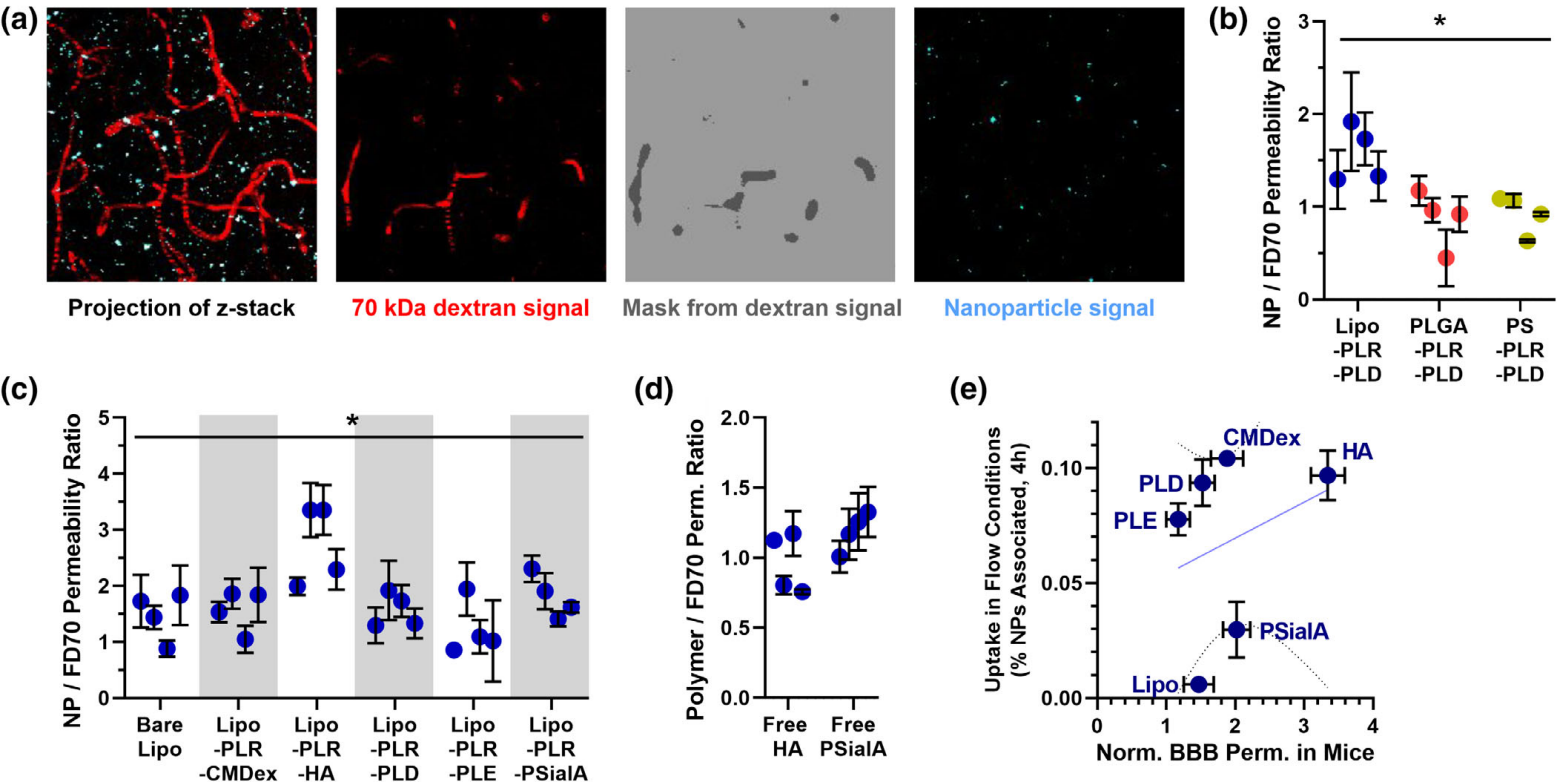
Intravital imaging enables the calculation of NP permeability across the blood–brain barrier (BBB) in mice. (a) From sequential z‐stack images, the starting signal for a 70 kDa FITC‐dextran (FD70) blood vessel marker is used to create a mask separating vessel pixels from brain parenchyma. This mask is applied to both dextran and nanoparticle signal for the first and all subsequent time points, allowing for calculations nanoparticle transport out of the vessels and into the brain. (b) For PLD outer layer nanoparticles with differing cores, liposome‐based materials displayed slightly higher BBB permeability than stiffer polymer particles. (c) For liposome core particles, an HA outer layer appeared to confer an advantage to crossing the blood brain barrier compared to other outer surface layers. (d) However, size‐matched HA and PSialA polymers did not cross the blood brain barrier faster than the biologically inert dextran. (e) Repeating the monolayer association assay, now with flow of nanoparticle treatments over the cell surface, better captured the high BBB permeability behavior of HA‐coated LbL‐NPs. Permeability data display mean ± standard error for individual animals, based on 3–15 permeability measurements, depending on length of experiment viability. * Treatment groups are statistically different (*p* < 0.05) by Kruskal–Wallis *H* test. Uptake data display mean ± standard deviation for six technical replicate channels in series on one flow chip. Trendlines display simple linear regressions of the data, and dotted lines indicate the 90% confidence interval of the trendline.

Having investigated the role of core stiffness and surface chemistry on uptake and transport of NPs by BBB endothelial cells in vitro, we next sought to understand the extent to which these controlling factors are recapitulated in vivo. To do so, we used a select set of LbL‐NPs (Supplementary Table 2) to examine the BBB permeability via intravital imaging through a cranial window in mice. This method has been used in previous studies to construct static images of NP accumulation in brain,^53^ and has recently been adapted to capture a time series of three‐dimensional images for quantitative permeability measurements between the brain capillaries and parenchyma.^22^ Briefly, fluorescent dextran and fluorescent NPs are administered intravenously, then a cranial window is generated, and two‐photon microscopy is used to generate a time series of multiphoton confocal images across the intact dura. The dextran signal from the first imaging time point is converted to a three‐dimensional mask to differentiate blood vessels from brain parenchyma, and the subsequent images are compared to this mask to determine the permeability of the NPs across the BBB (Figure 6a). As a passive diffusion marker, the consistent and slow leakage of high molecular weight dextran out of the blood vessels was also computed to use as a normalization factor, eliminating effects of any slight Z‐shifts over time that could not be accounted for by differential slice selection.

After validating that retroorbital NP injection yielded comparable results to the previously published method of tail vein injections (Supplementary Figure 5), we used this technique to determine the BBB permeability of LbL‐NPs in mice; all tabulated permeability values are available in Supplementary Table 3. For a constant PLD outer layer, compliant liposome‐based NPs had higher permeability than PLGA and PS core LbL‐NPs (Figure 6b), although the difference was less stark than in the in vitro assays. Similarly, comparing liposome core LbL‐NPs, varying surface chemistry generally had a modest impact on BBB permeability (Figure 6c), and for most of the polyanion outer layers studied here, LbL functionalization did not increase NP transport over bare liposomes, consistent with observations from the transwell transport assay. Unexpectedly, we observed that HA‐coated NPs did significantly out‐perform all other formulations in vivo, including bare NPs and other carboxylated carbohydrates CMDex and PSialA. HA is a known binding partner for several receptors and other proteins—including CD44 and HA binding proteins (HABPs)—which are expressed by brain endothelial cells, including hCMEC/D3^35^ (Supplementary Figure 6); however, these binding partners have not been implicated in transcytosis or other uptake activity in endothelial cells.^54^

We hypothesized that the HA‐coated NPs were undergoing random binding events with endothelial cell surface binding partners as they passed through the capillaries. This phenomenon would slow transit of NPs—known to localize poorly to blood vessel walls^55^—in the blood flow environment and enhance their localization to the BBB surface, allowing for a higher probability of nonspecific uptake by macropinocytosis, as was implied from our in vitro microscopy analysis. To probe this, we generated Cy5‐labeled HA and PSialA polymers, size matched to the FD70 diffusion marker, and examined their BBB permeability in vivo (Figure 6d). Neither polymer had higher permeability than the passively diffusing dextran, thus we concluded that the polymer interactions with cell surfaces do not, on their own, drive active uptake and transcytosis. Rather, we posit that a combination of the adsorbed polymer morphology and its presentation on the NP surface with the flow conditions of the capillaries is required to convey a transport benefit. To further probe this behavior, we employed flow chips to construct a lower‐throughput but dynamic version of the monolayer association assay reported in Figure 4d, with pump‐driven flow of NP treatments parallel to the apical surface of the hCMEC/D3 monolayers grown on collagen‐coated plasticware. Treatments were flowed over the cells at 0.79 ml/min—1 dyne/cm^2^ shear stress to mimic the low end of the highly heterogeneous shear range in the brain vasculature^56^ while avoiding undue stress to statically grown cells—for 4 h. The resulting cell uptake of NPs (Figure 6e, with a comprehensive set of cross‐comparisons displayed in Supplementary Figure 7) matched well with measured NP permeability in flowing capillaries in vivo*—*including the identification of HA‐coated NPs as a top candidate—but overpredicted the permeability for CMDex‐, PLD‐, and PLE‐coated NPs compared to the in vivo permeability measurements. While this further supports our hypothesis that HA coatings improve NP permeability by changing their flow behavior at the BBB, it also underscores our finding that no single in vitro metric in our current toolbox can completely and accurately predict nanomaterial uptake across the BBB in vivo. Further, it highlights that researchers must be conscious of how the model that they pick may affect decisions to move forward with particular formulations.

## 3 MATERIALS AND METHODS

### 3.1 Materials

Here, 1,2‐distearoyl‐sn‐glycero‐3‐phospho‐(1′‐rac‐glycerol) sodium salt (DSPG), 1,2‐distearoyl‐sn‐glycero‐3‐phosphoethanolamine (DSPE), 1,2‐distearoyl‐sn‐glycero‐3‐phosphocholine (DSPC), and cholesterol were purchased from Avanti. Sulfo‐cyanine dyes with NHS ester or amine handles were purchased from Lumiprobe. Chloroform was purchased from TCI. Methanol, Poly(d,l‐lactide‐glycolide) (PLGA Resomer RG 502H, 7–17 kDa), rhodamine B—PLGA (50:50 monomer ratio, 10–30 kDa), the hCMEC/D3 cell line, Accumax dissociation reagent, Type 1 rat‐tail collagen, ascorbic acid, β‐mercaptoethanol, LY, FITC‐labeled dextrans, DMSO, heparin sulfate, bovine serum albumin (BSA), saponin, and sulfo‐*N*‐hydroxysuccinimide (sulfo‐NHS) were purchased from Millipore Sigma. Cy5‐functionalized PLGA (10–15 kDa) was purchased from PolySciTech. Whatman Nucleopore polycarbonate hydrophilic membranes (400, 200, 100, and 50 nm sizes) were purchased from GE. 50/15 ml Falcon tubes, DNA LoBind tubes, 10% neutral buffered formalin, PS semi‐micro cuvettes, 0.22 μm polyethersulfone syringe filters, Spectrapor dialysis membranes, microscope slides, coverslips, slide sealer nail polish, Masterflex size 14 platinum‐cured silicone tubing, and polypropylene Masterflex fittings were purchased from VWR. D02‐E300‐05‐ND, 02‐E100‐05‐N, and C02‐E100‐05‐N TFF filters, as well as 15 ml reservoirs for peristaltic pump circuits, were purchased from Repligen. PLR hydrochloride (38.5 kDa), PLD (14 kDa), and PLE (15 kDa) were purchased from Alamanda Polymers. HA (40 kDa) was purchased from LifeCore Biomedical. CMDex (10–20 kDa) and PSialA (n = 8 to >100 residues) were purchased from Carbosynth. DTS 1070 folded capillary zeta cells were purchased from Malvern. Tissue culture plasticware (T75, T25, clear and white 96 well plates), 24‐well 1 μm pore transwell plates, individual transwell inserts, Penicillin/Streptomycin and fetal bovine serum (FBS) were purchased from Corning. EBM‐2 cell culture media was purchased from Lonza. Phosphate buffered saline (PBS), LabTek 8‐chamber coverslips, Hoechst 33342, fluorescently labeled wheat germ agglutinin, fluorescently labeled phalloidin, AlexaFluor 488‐labeled anti‐VE Cadherin (16B1), AlexaFluor 488‐labeled anti‐ZO‐1 (ZO1‐1A12), 5 M bioreagent grade NaCl solution, 1 M bioreagent‐grade HEPES, chemically defined lipid concentrate, basic fibroblast growth factor, PCR tube strips with caps, Pierce endotoxin removal columns, and yellow‐green or red fluorescent PS microspheres (100 nm Fluospheres) were purchased from Thermo Fisher. RNeasy Plus Mini Kits for RNA extraction and QuantiTect Reverse Transcriptase Kits were purchased from Qiagen. Roche Light Cycler‐DNA Master SYBR Green I mastermix and Corning Axygen 384‐well PCR microplates were purchased through the MIT BioMicro Center/KI Genomics Core. IDTE buffer, nuclease free water, and PrimeTime PCR Primers were purchased from Integrated DNA Technologies. The Voltohmmeter and accompanying electrodes were purchased from World Precision Instruments. 1‐Ethyl‐3‐[3‐dimethylaminopropyl]carbodiimide hydrochloride (EDC) was purchased from Chem‐Impex. Falcon cell strainer tubes were purchased from Fisher Scientific. Syntaxin 6 (C34B2) Rabbit mAb 2869, Caveolin‐1 (D46G3) XP Rabbit mAb 3267, Clathrin Heavy Chain (D3C6) XP Rabbit mAb 4796, EEA1 (C45B10) Rabbit mAb 3288, and Anti‐rabbit IgG (H + L), F(ab’)2 Fragment (Alexa Fluor 488 Conjugate) #4412 were purchased from Cell Signaling Technologies. Then, 6 mm biopsy punched were purchased from McKesson. VECTASHIELD Antifade Mounting Medium (H‐1000) was purchased from Vector Laboratories. MatTek 35 mm Dishes (No. 1.5 Uncoated Coverslip, 7 mm glass diameter) were purchased from Fisher Scientific. μ‐Slide VI 0.4 chips were purchased from Ibidi.

### 3.2 Nanoparticle synthesis and characterization

#### 3.2.1 Liposome synthesis

Cholesterol and lipid stocks were made in chloroform and methanol, then combined in round bottom flask at a mol ratio of 31 Chol:31 DSPC:7 DSPE:31 DSPG. The lipids were dried into a thin film using a BUCHI rotary evaporation system under heat (55°C, water bath) until completely dry (<30 mBar). A Branson sonicator bath was heated to 65°C, at which point the RBF with the lipid film was partially submerged in the bath and a volume of Milli‐Q deionized water was added to resuspend the lipid film to a 1 mg lipid/ml solution. The liposome solution was sonicated three times for (1 min on, 1 min off), then transferred to an Avestin LiposoFast LF‐50 liposome extruder. The extruder was connected to a Cole‐Parmer Polystat Heated Recirculator Bath to maintain a temperature of 65°C. The liposomes were extruded through nucleopore membranes until a 50–100 nm liposome was obtained. Typically, this was achieved by passing through stacked 400 and 200 nm membranes, 100 nm, and 50 nm membranes. The liposomes were analyzed by DLS to verify sizes less than 100 nm and PDI values less than 0.2. Fluorescently labeled liposomes were prepared via NHS‐coupling of Sulfo‐cyanine dyes with NHS ester handles to DSPE head group amines; reactions in 15 mM sodium carbonate buffer (pH 9), stirred at room temperature overnight. Unconjugated dye was purified away from the labeled liposomes by TFF before liposome characterization by DLS.

#### 3.2.2 PLGA nanoparticle synthesis

PLGA (10 mg) was dissolved at a concentration of 5 mg/ml in acetone, with a mass ratio of 9:1 Resomer 502H to dyed (RhodB or Cy5) polymer. Milli‐Q water (12 ml) was added to a scintillation vial and stirred gently on a stir plate while heating to 35°C. The PLGA solution was drawn up in a syringe with a 26‐gauge needle then slowly added to the water under constant stirring. An additional 8 ml of 35°C deionized water was added, and the vial was left to stir, uncovered under ventilation, for at least 2 h. Particles were characterized by DLS and concentrated to 1 mg/ml by TFF.

#### 3.2.3 LbL polymer functionalization

Nanoparticles—liposomes, PLGA NPs, or commercially purchased PS fluospheres—were layered with polyelectrolyte coatings by adding NP suspension (0.5 mg/ml when layering HA, 1 mg/ml otherwise, all in unbuffered water) to an equal volume of polyelectrolyte solution under sonication at room temperature.^57^ This process is highly scalable and, depending on the formulation at hand, ranged from 2 to 40 ml in total volume. The mixture was sonicated for approximately 3 s then vortexed at maximum speed for approximately 10 s. The weight equivalents (wt. eq.) of polyelectrolyte used with respect to liposome core were 0.4 for PLR, 1.6 for CMDex, 1.2 for HA, 0.8 for PLD, 3 for PLE, and 1.4 for PSialA. Polyelectrolyte solutions were prepared in 50 mM HEPES (pH 7.4) and 40 mM NaCl, with the exception of HA, which was prepared in 2 mM HEPES. The freshly layered particles were allowed to incubate at room temperature for 5–30 min, then purified using the TFF.

#### 3.2.4 Tangential flow filtration

To purify any unadsorbed polyelectrolytes at each step of layering, NP samples were connected to a Spectrum Labs KrosFlo II filtration system using Masterflex, Teflon coated tubing. D02‐E100/E300‐05‐N (batch volume ≥12 ml) or C02‐E100‐05‐N (batch volume <12 ml) filters with 300 kDa (HA and CMDex purifications) or 100 kDa (all other purifications) nominal molecular weight cutoffs were used to purify free dyes or polymers away from the NP samples. For PLR purification, the filter was pre‐treated using a mock sample of free PLR, so saturate adsorption sites on the anionic membrane walls. Samples were filtered at 13 ml/min for small batches or 80 ml/min for large batches, with a Milli‐Q water inlet line to replace 1:1 the volume of waste permeate. After at least five sample volume equivalents of waste collection, the sample was concentrated, removed from the filter by reversing the pump direction, and brought back to 1 mg/ml NP concentration (0.5 mg/ml for HA) by backflushing a defined volume of Milli‐Q water through the filter and into the sample.

#### 3.2.5 Nanoparticle characterization

Nanoparticle hydrodynamic size, polydispersity, and zeta potential were measured using DLS (Malvern ZS90 Particle Analyzer, *λ* = 633 nm, *θ* = 90°). Without additional sonication, 50 μg of each NP was diluted into 2 mM NaCl to give a total volume of 800 μl, then transferred to PS cuvettes or DTS1070 folded capillary cuvettes for DLS. Refractive indices and absorption coefficients for measurements were: liposome—1.450, 0.001; PLGA—1.560, 0.000; PS—1.590, 0.010. Dispersant used refractive index and viscosity values for water: 1.330, 0.8872 cP (at 25°C). Nanoparticle micrographs were acquired using TEM on a JEOL 2100F microscope (200 kV) under cryogenic conditions. Nanoparticle sizes from TEM images were counted manually using ImageJ.

### 3.3 Cell culture

#### 3.3.1 Maintenance

Here, hCMEC/D3 cells—immortalized endothelial cells derived from an adult female patient^11^—were cultured according to manufacturer specifications in EBM‐2 media supplemented with 5% FBS, 1 ng/ml bFGF, 1.4 μM hydrocortisone, 1% pen/strep, 1% chemically defined lipid concentrate, 10 mM HEPES, and 5 μg/ml ascorbic acid. Cells were cultured in flasks coated with 12 μg/cm^2^ rat‐tail collagen and split twice per week at a ratio between 1:3 and 1:8, using Accumax dissociation reagent, to maintain cells below approximately 90% confluency. Between maintenance and experiments, cells were incubated at 37°C and in a 100% humidity and 5% CO2 atmosphere. Cell lines were authenticated using STR profiling, and cells were tested monthly for mycoplasma, with all results coming back negative for contamination.

#### 3.3.2 Cell monolayers for gene expression and uptake experiments

All plasticware for cell studies was pre‐coated with rat‐tail collagen. hCMEC/D3 cells were suspended in media and seeded at a density of 2 × 10^5^ cells/cm^2^ transwell supports or 96‐well tissue culture plates. The cells were incubated for 7 days, with media replacement every 2–3 days. For transwells, the TEER was monitored to confirm proper barrier formation before use in any experiments. TEER is expressed as the resistance of transwell filters with cells minus transwell filters without cells.

### 3.4 Transport experiments

#### 3.4.1 General transwell transport protocol

Cell monolayers in transwells were transferred to 24‐well plates containing 1 ml/well media. Nanoparticle or fluorescent marker treatments were added to the apical chambers at 20 μg/cm^2^ in media; negative control wells received fresh media. Extra treatments for each experiment were used to make fluorescence calibration curves. At 4, 8, and 24 h after treatment addition, the monolayers were transferred to new basal plates (creating quasi‐sink conditions for the basal media compartment). Media from the basal plates was sampled for fluorescence measurements on a Tecan Infinite M200 Pro plate reader, with 100 μl/well sample in a black 96‐well plate, and applied to the calibration curves to calculate percent of nanomaterial transported.

#### 3.4.2 4°C protocol

To assess active transport, NP treatments in media and basal plates with media were both equilibrated to 4°C in the refrigerator. Pre‐experiment TEER values were measured with cells still at 37°C, then monolayers were transferred to the chilled treatment and basal media. The monolayers were then incubated in the 4°C refrigerator for the remainder of the experiment.

#### 3.4.3 DMSO breakup and reread

To homogenize samples, 100 μl (NPs in cell culture media) per well was supplemented with 100 μl/well DMSO and 50 μl/well of heparin sulfate (1 mg/ml in PBS). The plate was placed on an orbital shaker at 240 rpm for 10 min before repeating the fluorescence measurements.

### 3.5 Gene expression

#### 3.5.1 RNA extraction and cDNA preparation

Cells were rinsed with PBS and pelleted. Total RNA was extracted according to the instructions provided with the Qiagen RNeasy kit (available online as the RNEasy Mini Handbook). Briefly, lysis buffer was prepared with 1% β‐mercaptoethanol to protect RNA from degradation. Lysate samples of 1–3 million cells in 350 μl lysis buffer were mixed with 350 μl ethanol and spun through the filter columns. Columns were washed once with 700 μl RW1 buffer then twice with 500 μl RPE buffer. Total RNA was eluted from the columns using 30 μl nuclease‐free water. Nanodrop (Thermo Fisher) spectrophotometry was used to assess RNA concentration and quality, and all 260/280 values were greater than 1.8. cDNA was synthesized according to manufacturer's instructions using 1 μg of template RNA. cDNA was stored at −20°C or placed on ice for immediate use.

#### 3.5.2 Real‐time quantitative PCR

For qPCR reactions, cDNA was diluted 1:50 with nuclease‐free water, and primers were diluted to 20x (10 μM) in IDTE buffer according to the manufacturer's specifications. RT‐qPCR was set up in a 384‐well plate with 8 μl diluted cDNA, 10 μl 2x SYBR Green master mix, 0.8 μl nuclease‐free water, and 1.2 μl 20x primer. Each condition was performed in technical triplicate. No primer (IDTE buffer instead of primer) and no cDNA (water instead of cDNA) controls were also used to ensure there was not contamination. RT‐qPCR was run on a LightCycler 480 (Roche) and Ct values obtained using the second derivative. The ΔCt method was used to compare expression between cell lines, normalizing to beta actin. The primers have the following assay ID numbers and sequences:

**Table:** 

Gene	Code	IDT Assay #	Primer 1 sequence	Primer 2 sequence
Beta actin	ACTB	Hs.PT.39a.22214847	CCTTGCACATGCCGGAG	ACAGAGCCTCGCCTTTG
Transferrin receptor	TFRC	Hs.PT.39a.22214826	CCCAGTTGCTGTCCTGATATAG	TCTGGATAAAGCGGTTCTTGG
LDL receptor related protein 1	LRP1	Hs.PT.58.40048480	TCCAGTACAGATTGTCTCCCA	ATCTACTTTGCCGACACCAC
Zonula occludens 1	TJP1	Hs.PT.58.2456962	GCTGGCTTATTCTGAGATGGA	CGCGTCTCTCCACATACATTC
VE‐cadherin	CDH5	Hs.PT.58.4732035	TGCCCACATATTCTCCTTTGAG	GAACCAGATGCACATTGATGAAG
CD34	CD34	Hs.PT.56a.24708916	TGCCTGAACATTTGATTTCTGC	GACCTTTCAACCACTAGCACT
Von Willebrand factor	VWF	Hs.PT.58.40497129	TGACCTTGCAGAAGTGAGTATC	GTTGTGGGAGATGTTTGCCTA
Platelet endothelial cell adhesion molecule	PECAM1	Hs.PT.58.19487865	ATTGCTCTGGTCACTTCTCC	CAGGCCCCATTGTTCCC
CD44	CD44	Hs.PT.58.3636696	GATCACCGACAGCACAGAC	TTGCTCCACCTTCTTGACTC
Hyaluronan binding protein 1	HABP1	Hs.PT.56a.20789542	GAAGCGAAATTAGTGCGGAAAG	TCCTGTTCTTCAACCTTCTGC

### 3.6 In vitro confocal imaging

#### 3.6.1 Fixed sample treatment and preparation

For fixed cell imaging of structural proteins and NP localization within intracellular compartments, cells were cultured on collagen‐coated chambered slides or transwells for 7 days to allow complete monolayer development. The cells were incubated with Cy3‐labeled NPs for 8 h, then washed 3x with PBS and fixed with 4% formaldehyde in PBS for 20 min at room temperature, protected from light. This was followed by three washes with PBS at 5 min/wash, and samples were stored at 4°C overnight in fresh PBS. Samples were permeabilized and blocked with 0.1% saponin and 1% BSA in PBS for 30 min at room temperature. For samples with unlabeled primary antibodies, cells were stained for 30 min with primary solutions containing 0.1% saponin, 1% BSA, and 2.5 μg/ml primary antibodies. The samples were rinsed three times with PBS over the course of 15 min, then stained with solutions containing 2.5 μg/ml AlexaFluor 488 labeled antibodies, 1.5 μg/ml Hoechst 33342, and either 10 μg/ml wheat germ agglutinin or 0.165 μM phalloidin labeled with AlexaFluor 633. The samples were washed again with PBS three times over 15 min, then plastic chambers were removed from chamber slides, or transwell filters were punched out of their plastic casing using a biopsy punch and placed cell side up on a microscope slide. The samples were supplemented with Vectashield (H1000), secured with a coverslip, and sealed using nail polish. The samples were stored at 4°C and protected from light until imaging.

#### 3.6.2 Live sample treatment and preparation

For live cell imaging of dextran macropinocytosis, were cultured on collagen‐coated (12 μg/cm^2^) MatTek dish glass coverslips for 7 days. The cells were incubated with 20 μg/cm^2^ each Cy3‐labeled NPs and 70 kDa FITC‐Dextran for 8 h, then washed 3x with fresh media. After adding fresh (HEPES‐containing) media containing 10 μg/ml, the dishes were sealed using parafilm, and the live cells imaged immediately.

#### 3.6.3 Image capture and processing

All cells were imaged with a confocal laser‐scanning microscope (FV‐1200, Olympus), equipped with 405, 473, 559, and 635 nm lasers. Images were acquired with 100x objectives, and all images were acquired under the same illumination settings. Images were processed using ImageJ software.

For published images, bare liposome signal was linearly increased by 10%, and Syntaxin 6 background signal was reduced by raising the lower pixel limit by 10%. No other adjustments to image signals were made. With the exception of orthogonal views, images display Z projections (maximum signal) bounded by the center of the cell layer and the top of all four signals at the apical surface.

Mander's coefficients for NP signal overlap with intracellular markers was calculated on unadjusted images using Imaris 3D software. Image stacks were trimmed to remove and discard slices outside of the cell layer, then thresholded using the built‐in Costes^58^ Automatic Thresholding plugin.

### 3.7 Flow cytometry

To assess NP association at single‐cell resolution, particle‐treated cells in 96‐well plates were washed 3x with warm PBS, then dissociated from the well bottoms using 30 μl Accumax dissociation reagent. Then, 220 μl warm media was added to quench the dissociation, and pipetted vigorously to break up clumps before transferring to new 96‐well plates without collagen coating. The samples were analyzed using a BD LSR II Flow Cytometer with a high throughput sampler (BD Biosciences). Samples dosed with Cy5 liposomes were analyzed on the APC channel (e.g., 640, filters 670/30). Samples dosed with rhodamine B PLGA were analyzed on the PE channel (e.g., 561 filters 582/42). Samples dosed with yellow green (PS) fluospheres were analyzed on the GFP channel (e.g., 488, filters 515/20). Data were analyzed using FlowJo (version 10), and cells were gated for single cells based on untreated cell samples using the side scatter and forward scatter plots.

### 3.8 Monolayer association

To assess NP association at the population level, NP treated cells in 96‐well plates were washed three times with ice cold PBS, then treated with 100 μl/well of 0.5 mg/ml heparin sulfate in 50% DMSO and 50% PBS. Standard curves were constructed by adding defined amounts of NPs to the same solution. Samples were placed on an orbital shaker for 15 min at 180 rpm, then transferred to black 96‐well plates to measure particle fluorescence.

### 3.9 IBIDI flow chip association

To assess NP association with cells under fluid flow conditions, hCMEC/D3 cells were seeded in 60 μl media at a density of 2 × 10^5^ cells/cm^2^ in collagen‐coated, 6‐channel Ibidi μ‐Slide VI 0.4 chips, and each port supplemented with 60 μl media to total 180 μl of media per channel. Media was static during the development phase, and changed every 2–3 days for 7–8 days. Developed cells were treated with NPs at 7.2 μg/ml, 10 ml total (to give 20 μg/cm^2^ total) continuously flowing in series through all six channels on one chip. Flow was driven through a closed system loop by a peristaltic pump through sterilized Masterflex platinum‐cured silicone tubing—size 14—at 0.79 ml/min (1 dyne/cm^2^ shear stress) for 4 h, with a sterile air filter attachment preventing buildup of pressure differential along flow path. Treatments were removed, and the channels were rinsed three times with 180 μl PBS. Each channel was treated with 100 μl/well of 2 mg/ml heparin sulfate in 75% DMSO and 25% PBS for 30 min, cycling 60 μl within each channel every 10 min. Standard curves were constructed by adding defined amounts of NPs to the same homogenization solution. The samples and standards were transferred to black 384‐well plates to measure particle fluorescence using the plate reader.

### 3.10 Animal studies

#### 3.10.1 Animal care and use

All animal experiments were approved by the Massachusetts Institute of Technology Committee on Animal Care (CAC, protocol number 0919‐056‐22) and were conducted under the oversight of the Division of Comparative Medicine (DCM). C57BL/6 mice were purchased from Taconic, and were housed in cages of no more than five animals with controlled temperature (25°C), 12 h light–dark cycles and free access to food and water. Both female and male mice were used in this study, and the mice were 8–20 weeks old at the time of experiment. The free‐to‐use PS power calculator (Vanderbilt)^59^ was used to determine the minimal sample size for which statistical power was greater than or equal to 0.8, leading to groups of two female + two male mice, with one to three image sets per animal.

#### 3.10.2 Intravital imaging

Mice underwent head hair removal up to 24 h before the imaging procedure occurred. All surgical tools were one‐time‐use sterile products or sterilized in an autoclave prior to surgery. One at a time, animals were anesthetized via a 100 μl intraperitoneal injection of 100 mg/kg ketamine with 10 mg/kg xylazine paralytic carried in sterile PBS. Once fully anesthetized (unreactive to toe pinch stimulus), mice were injected with 70 kDa FITC‐labeled dextran (2 mg/ml in PBS, sterile filtered) and red‐fluorescent NPs (1 mg/ml in 5% dextrose), both as 150 μl retro‐orbital injections. To create the cranial window, the skull was exposed, and a high‐speed hand drill (Dremel) was used to thin the skull until the dura mater was exposed over the right frontal cortex. The mice were then secured to a microscope coverslip for imaging. Multiphoton imaging was performed on an Olympus FV‐1000MPE multiphoton microscope (Olympus) using a 25×, numerical aperture 1.05 objective. Excitation was achieved by using a femtosecond pulse laser at 840 nm, and emitted fluorescence was collected by photomultiplier tubes with emission filters of 425/30 nm for Collagen 1, 525/45 nm for fluorescein isothiocyanate‐labeled dextran, 607/45 nm for red PS NPs, and 672/30 nm for Cy5 (PLGA and liposome) NPs. Collagen 1 was excited by second harmonic generation and emitted as polarized light at half the excitation wavelength. The collagen 1 signal was used to identify the dura, such that the vessels imaged were within the cortex.^22^ Images were acquired every 2 min for 12 min for analysis, and up to three image sessions per mouse were run. Mice were maintained under anesthesia by an additional, 100 μl intraperitoneal dose of 50 mg/kg ketamine in sterile PBS, 35–40 min after the first injection of anesthetic. Animals were euthanized by cervical dislocation directly from anesthetic sleep, complying with American Veterinary Medical Association (AVMA) guidelines.^60^

Acquired images from intravital imaging were then thresholded and segmented by using the Fiji distribution of ImageJ and the Trainable Weka Segmentation plugin. Vessels below the dura and arteries were examined to ensure that these represent BBB capillaries in the mouse brain. The microvasculature filled with dextran (dextran channel) was used to generate a three‐dimensional mask of the BBB mouse vessels. This mask was employed to calculate vessel surface area, as well as dextran and NP signal both inside and outside the blood vessels. After masking, analysis of NP or dextran transport was performed as previously described.^22^, ^29^, ^61^

### 3.11 Fluorescent polymer modifications

To create polymers that could be tracked in fluorescent microscopy, HA (60 kDa nominal molecular weight) and PSialA were dyed for intravital imaging using standard EDC coupling with amine‐functionalized SulfoCy5. Polymers, sulfo‐NHS, sulfoCy5‐amine, and EDC were added sequentially in a 1 monomer:0.5:0.255:2.55 molar ratio for HA, or 1 monomer:0.5:0.085:1.5 molar ratio for PSialA. Reactions were allowed to run overnight, then the polymers were purified away from free dye and reactants using Amicon Ultra‐4 3 kDa spin filters, followed by dialysis in Milli‐Q water using 3.5 kDa cutoff tubing. Dialysis continued until no dye could be detected in the dialysate.

### 3.12 Statistics

Detailed statistical information is provided for each figure in the associated caption, and all tests used nonparametric comparisons based on sample sizes. Unless noted otherwise, for single comparisons, the Mann–Whitney test was used. For multiple comparison testing, the Kruskal–Wallis test was used. Mann–Whitney and Kruskal–Wallis statistics were calculated using standard arithmetic formulas in Microsoft Excel 2021, while arithmetic means, standard deviation, standard error of the mean, and 90% confidence intervals were calculated and plotted by GraphPad Prism Version 10.0.1.

## 4 CONCLUSIONS

In this work, we have comprehensively characterized the uptake and transport of a combinatorial NP library in four in vitro BBB assays, and compared these results directly with in vivo permeability in live mice. Our data highlight major differences between in vitro metrics of nanomaterial uptake/association versus transport at the endothelial barrier of the BBB. hCMEC/D3, the most commonly used human immortalized cell line for BBB modeling, expresses tight junctions that do not recapitulate BBB exclusion of small molecules, but do provide an appropriate barrier for studying interactions with nanoscale materials. We demonstrated that cell assay behavior and gene expression are consistent across a range of commonly used development times and cell culture substrates, allowing for cross‐comparison with assays in the literature. For the three most common in vitro metrics of nanomaterial uptake and transport—flow cytometry, monolayer association, and transwell transport—flow cytometry provides the best correlated data to liposome‐based NP uptake studies in vivo. However, monolayer association and transwell transport allow for better cross‐comparison across different NP cores in LbL‐NP formulations. Across our library of 18 NPs, we show that the NP core composition consistently has the greatest effect on total uptake, with softer liposome cores undergoing the greatest magnitude of NP uptake or transport. In contrast, surface chemistry is a stronger determinant of intracellular trafficking patterns such as lysosomal versus transcytosis routing, which is highly relevant for drug delivery applications. We show that surface chemistry determines whether an NP is transported across the BBB intact or degraded in lysosomes, a finding that was only captured using transwell transport. The observations on the relative importance of LbL‐NP core stiffness and surface chemistries were conserved for permeability across the mouse BBB as measured via intravital imaging, though none of the static in vitro models predicted HA functionalization to have uniquely high permeability in vivo. This apparent advantage was partially captured by the in vitro flow system, but was not exclusive to HA in this setting, and the possibility remains that part of this discrepancy derives from differences in species differences between the human cells and the mouse BBB. Taken together, these data underscore that, while transwell transport gives the most complete picture of NP behavior in BBB endothelial cells, static in vitro uptake assays alone are not sufficient to identify top candidates for NP drug delivery carriers to the brain. While models of the singular BBB endothelial cell type allow higher throughput screening than co‐cultures, they necessarily will lack the contributions to drug delivery barrier properties provided by astrocytes, pericytes, and the extracellular matrix of the BBB. Instead, these in vitro metrics must be combined with a careful mechanistic understanding of how nanomaterials interact with endothelial cells—in both static and flow conditions, as well as without and with supporting cells—and judicious use of preclinical in vivo models, to provide comprehensive evidence for clinical translational potential.

## AUTHOR CONTRIBUTIONS

**Nicholas G. Lamson:** Conceptualization (lead); formal analysis (lead); investigation (lead); methodology (lead); project administration (lead); visualization (lead); writing – original draft (lead); writing – review and editing (equal). **Andrew J. Pickering:** Formal analysis (supporting); investigation (supporting); visualization (supporting); writing – review and editing (equal). **Jeffrey Wyckoff:** Investigation (supporting); methodology (supporting); resources (equal); writing – review and editing (equal). **Priya Ganesh:** Investigation (supporting); writing – review and editing (equal). **Elizabeth A. Calle:** Investigation (supporting); methodology (supporting); resources (equal); writing – review and editing (equal). **Joelle P. Straehla:** Funding acquisition (supporting); methodology (supporting); supervision (supporting); visualization (supporting); writing – review and editing (equal). **Paula T. Hammond:** Conceptualization (supporting); funding acquisition (lead); project administration (equal); supervision (lead); writing – original draft (supporting); writing – review and editing (equal).

## CONFLICT OF INTEREST STATEMENT

P.T.H. is a co‐founder and member of the board of LayerBio, a member of the Scientific Advisory Board of Moderna, and a member of the Board of Alector, Advanced Chemotherapy Technologies, and Burroughs‐Wellcome Fund. All other authors report no competing interests.

### PEER REVIEW

The peer review history for this article is available at https://www.webofscience.com/api/gateway/wos/peer-review/10.1002/btm2.10636.

## Supporting information


**DATA S1:** Supporting Information.

## Data Availability

No new materials or code were generated during this work. The data that support the findings of this study are available from the corresponding author upon reasonable request to the corresponding author.
