# Supplementary material for: Trafficking through the blood–brain barrier is directed by core and outer surface components of layer‐by‐layer nanoparticles
Source: Bioeng Transl Med. 2023 Dec 28;9(4):e10636. doi: 10.1002/btm2.10636 (PMC11256136; doi:10.1002/btm2.10636)
Supplement: Supplementary file 1 — DATA S1: Supporting Information. [file BTM2-9-e10636-s001.docx]

**Core material and surface chemistry of layer-by-layer (LbL) nanoparticles independently direct uptake, transport, and trafficking in preclinical blood-brain barrier (BBB) models**

**Authors:** Nicholas G. Lamson^1^, Andrew J. Pickering^1,2^, Jeffrey Wyckoff^1^, Priya Ganesh^1,3^, Elizabeth A. Calle^1,4^, Joelle P. Straehla^1,5,6^, and Paula T. Hammond^1,2,7,8,^*

^1^ Koch Institute for Integrative Cancer Research, Massachusetts Institute of Technology, Cambridge, MA 02142, USA.

^2^ Department of Chemical Engineering, Massachusetts Institute of Technology, Cambridge, MA, 02142, USA.

^3^ Department of Materials Science and Engineering, Massachusetts Institute of Technology, Cambridge, MA, 02142, USA

^4^ Department of Surgery, Massachusetts General Hospital, Boston, MA 02114, USA.

^5^ Department of Pediatric Oncology, Dana-Farber Cancer Institute, Boston, MA 02115, USA.

^6^ Division of Pediatric Hematology/Oncology, Boston Children’s Hospital, Boston, MA 02115, USA.

^7^ Broad Institute of MIT and Harvard, Cambridge, MA 02142, USA.

^8^ Institute for Soldier Nanotechnologies, Massachusetts Institute of Technology, Cambridge, MA 02139, USA.

* Correspondence to [hammond@mit.edu](mailto:hammond@mit.edu). 500 Main Street (76-553); Cambridge, MA 02136; (617) 258-7577

**Liposomes Lipo-PLR-PLD**


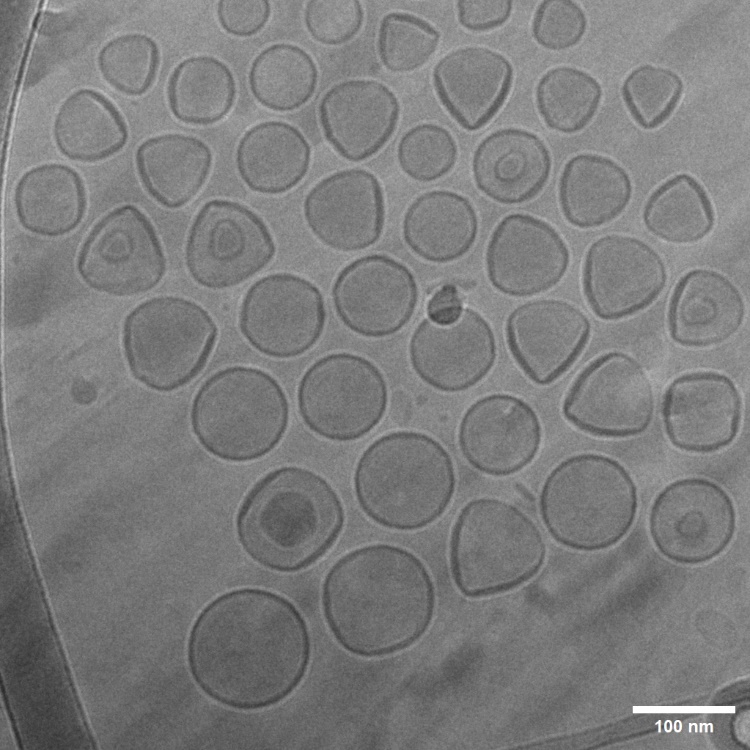




| **Formulation** | **DLS Number Average (d.nm)** | **DLS Z-avg (d.nm)** | **DLS PDI** | **TEM diameter ± standard dev** | **TEM # of NPs counted** |
| --- | --- | --- | --- | --- | --- |
| **Liposome** | 82.9 | 105.2 | 0.11 | 78.8 ± 18.3 | 116 |
| **Lipo-PLR-PLD** | 88.2 | 124.9 | 0.20 | 82.4 ± 16.4 | 107 |

**Supplementary Figure 1: Cryo-TEM analysis of LbL-NPs.** Diameters of nanoparticles in cryo-TEM micrographs were determined by manual measurement using ImageJ, then compared to sizes and polydispersity index as measured by dynamic light scattering. Scale bars display 100 nm.

**
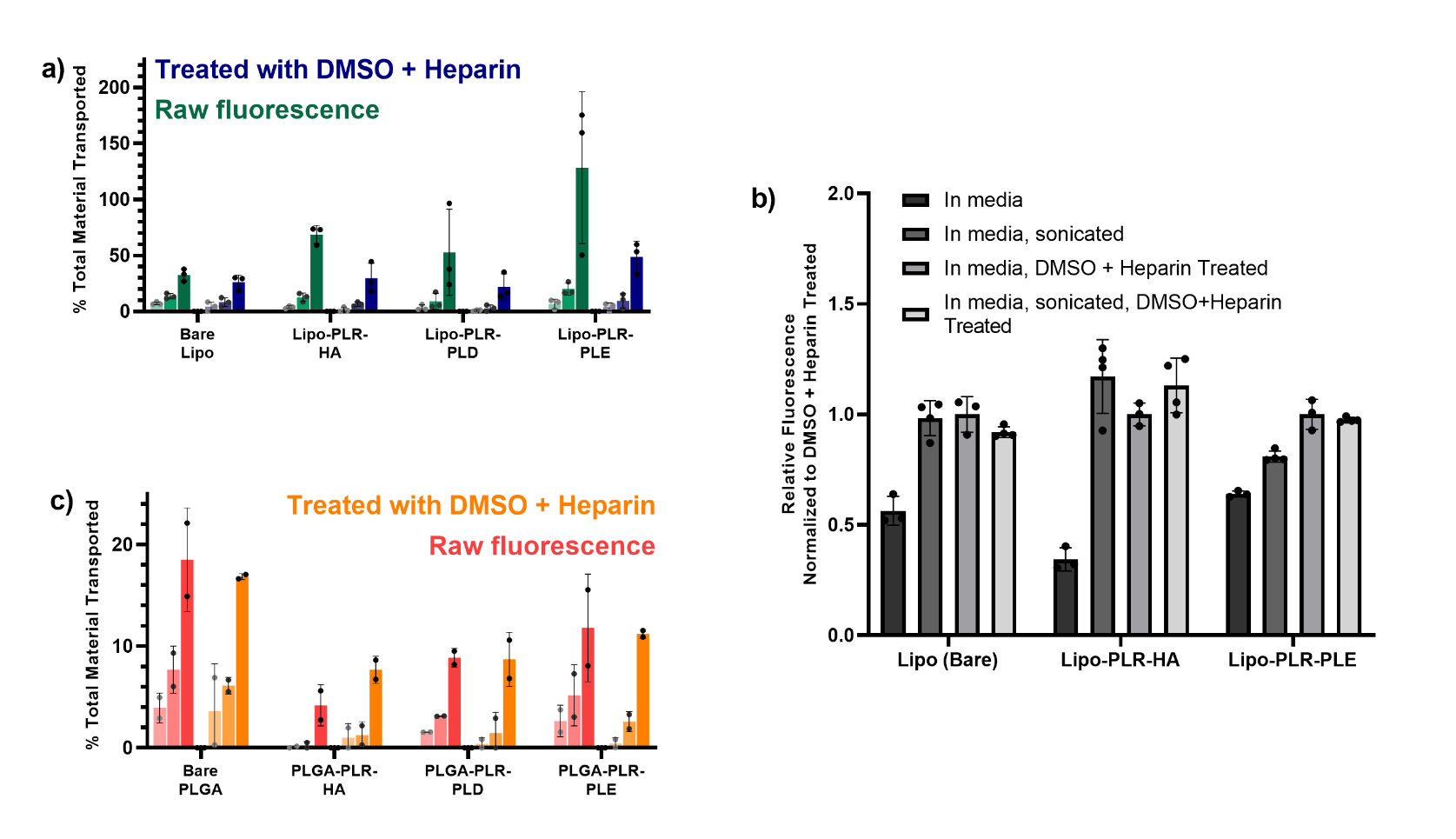
**

**Supplementary Figure 2: Liposome transport data in transwell models can be skewed by Cy5 fluorophore dequenching upon nanoparticle breakup. (a)** In a selection of liposome-based LbL-NPs, some outer layers – especially PLE – demonstrated drastic differences in transwell transport data depending on whether samples were treated with DMSO and heparin to break up NPs. **(b)** Nanoparticle fluorescent signal for all three formulations in cell culture media increased substantially and to approximately the same values after being broken up by sonicating, treating with DMSO + heparin sulfate, or both. Particle fluorescence data are normalized to samples treated with DMSO + heparin without sonication, and error displays standard deviation of 3 replicate wells of each sample. **(c)** By contrast, PLGA core LbL-NPs – which incorporate a rhodamine B fluorophore in quantities that do not self-quench – did not show these discrepancies. All transwell transport is reported as arithmetic mean ± standard deviation of three plate replicates, with three technical replicate wells per treatment per plate.

**
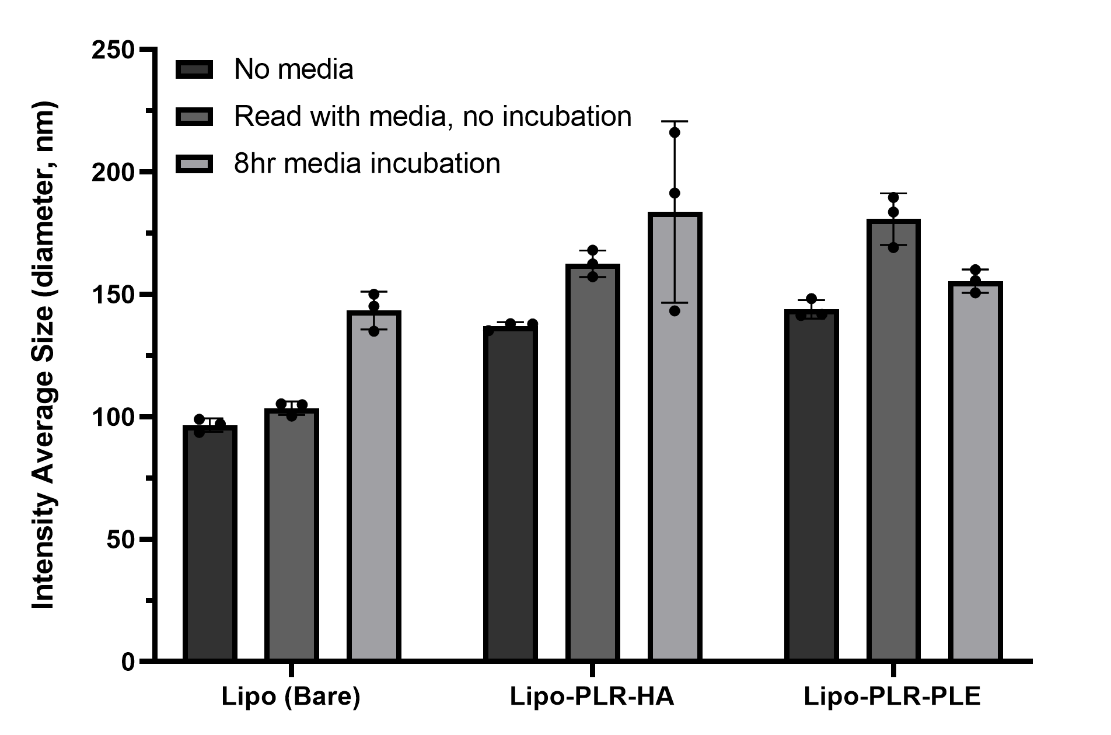
**

**Supplementary Figure 3: Nanoparticles do not substantially aggregate when incubated in cell culture media.** Nanoparticles were incubated in cell culture media for 8 hours at 37°C, then diluted into deionized water to measure size by DLS. Compared to control samples diluted in 2 mM NaCl or in 10% media (diluted in Milli-Q water) immediately before measuring, particles incubated in media displayed small changes in size consistent with protein adsorption but not particle aggregation. Data display mean ± standard deviation of three DLS measurements, and diameters are expressed as intensity average – rather than number average – as a metric that is more sensitive to smaller populations of particle aggregates.

**
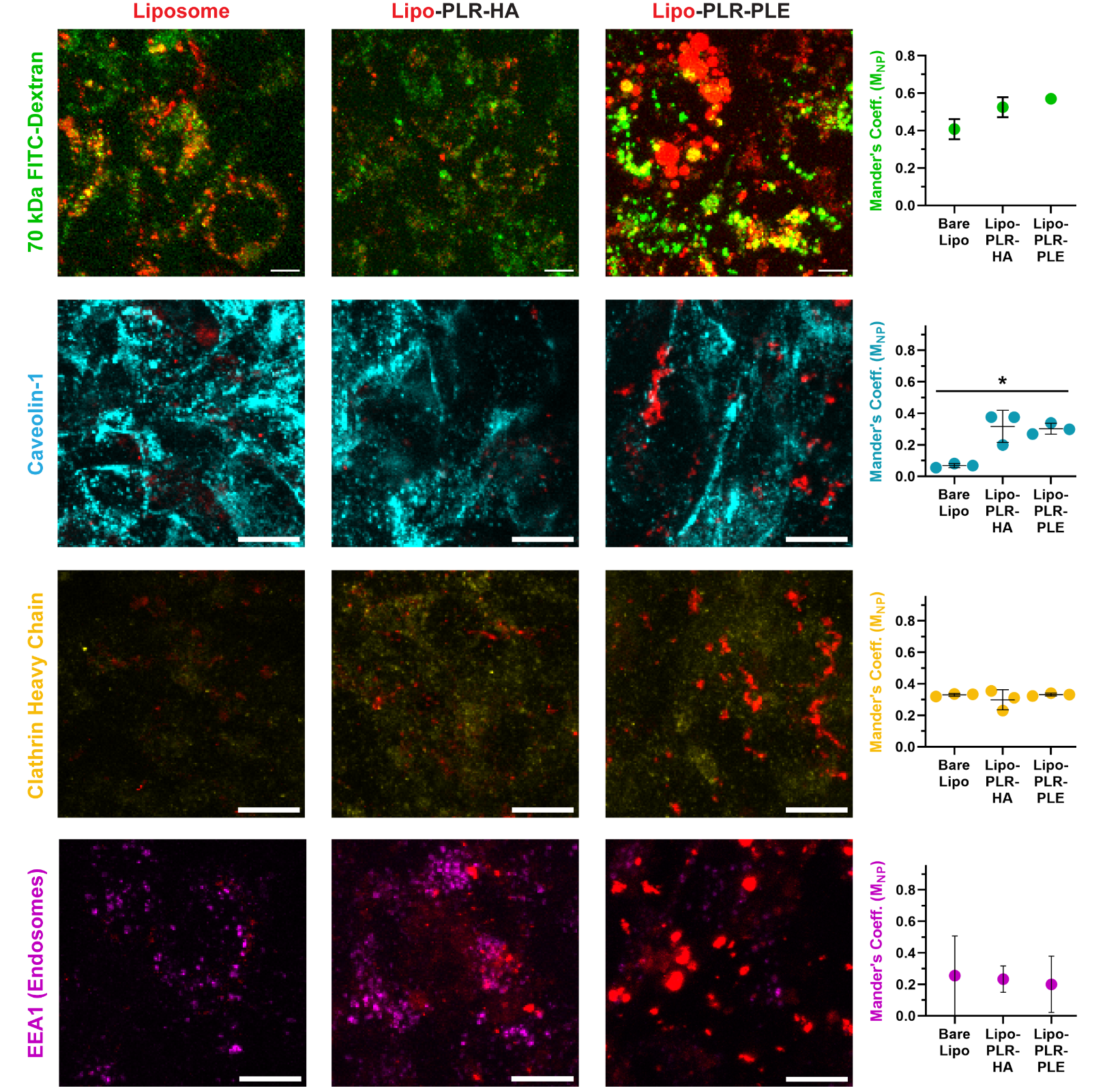
**

**Supplementary Figure 4: Nanoparticle uptake into endothelial cells appears to be mostly driven by non-specific mechanisms.** As a marker for nonspecific uptake – primarily macropinocytosis – a 70 kDa FITC-dextran demonstrated high overlap with the greatest fraction of signal for each nanoparticle. Nanoparticle signal for all three formulations did not colocalize strongly with immunofluorescence signal for protein machinery for caveolin or clathrin mediated endocytosis, with a particularly low correlation between liposome uptake and caveolin. Likewise, none of the nanoparticles demonstrated strong association with the EEA1 marker of early endosomes, suggesting that processing through this stage occurs quickly**.** Scale bars display 10 µm. Data display averages of three technical replicate images on three replicate monolayers from different hCMEC/D3 cell populations. Dextran and EEA1 data display one biological replicate due to experimental burden of live cell imaging (dextran) or inability to automatically threshold several sets of monolayer data (EEA1). * Treatment groups are statistically different (p < 0.05) by Kruskal–Wallis *H* test.

**
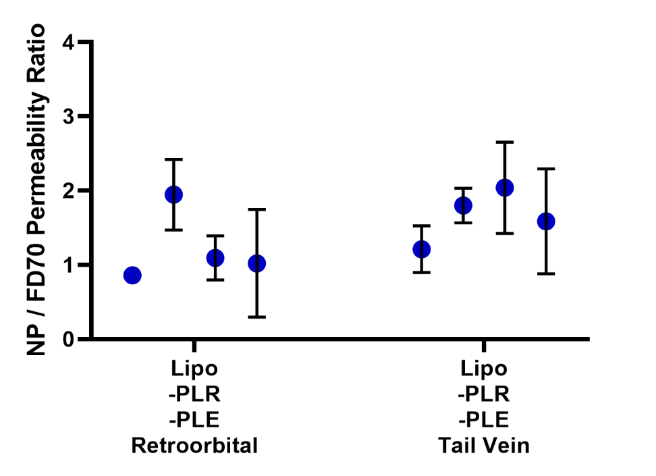
**

**Supplementary Figure 5: Retroorbital and tail vein injections yield comparable blood brain barrier permeability data for intravenously administered, fluorescent nanoparticles.** Data display mean ± s.e.m. for individual mice (n = 3-15 permeability measurements per mouse). Groups are not statistically significant at the p < 0.05 level by Mann-Whitney *U* test.

**
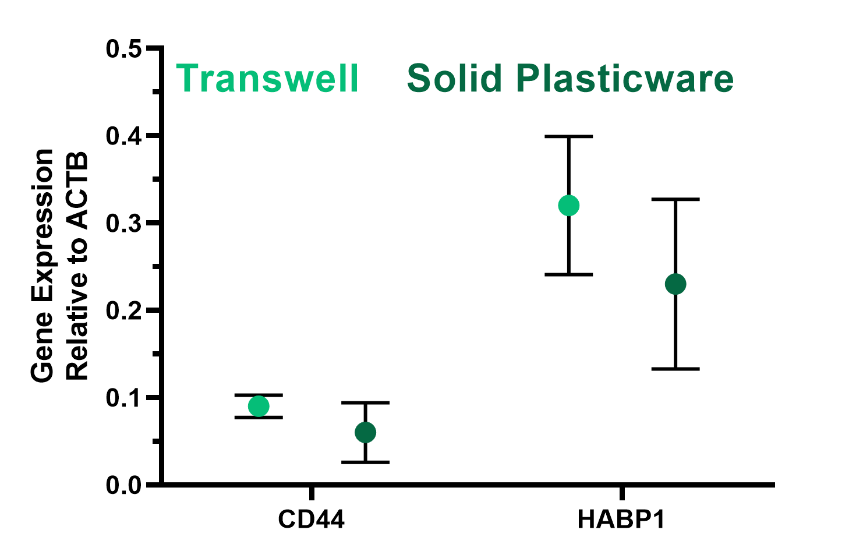
**

**Supplementary Figure 6: hCMEC/D3 cells express CD44 and HA binding protein 1 (HABP1) by qPCR analysis.** Cells were analyzed after 7 days of development post-seeding, and error bars display standard deviation of three biological replicates assayed at passage numbers 2, 6, and 10.


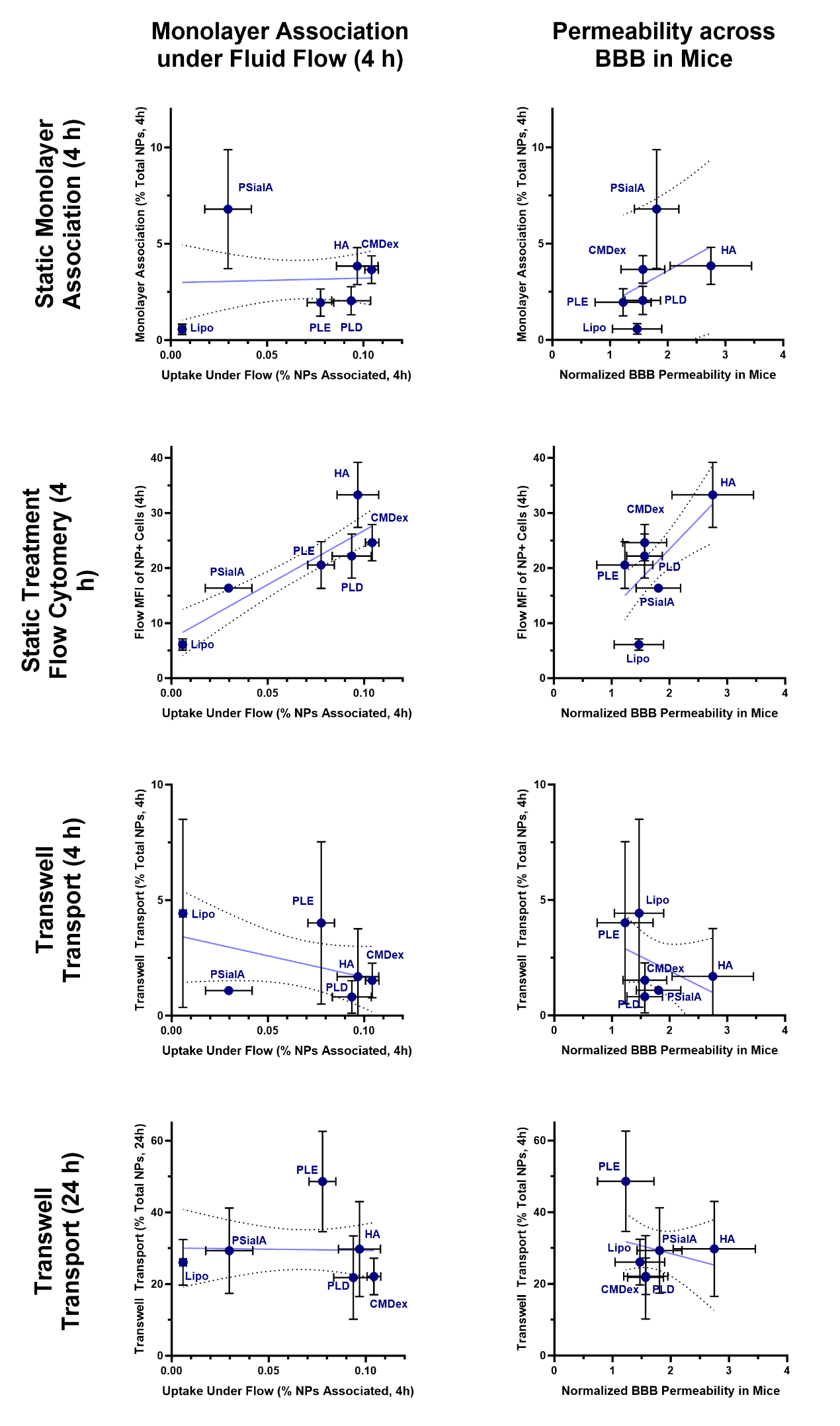
**Supplementary Figure 7: Comparison of uptake and transport metrics reveals varying degrees of predictive power for liposomal core LbL-NPs.** NP uptake as assayed by monolayer association or flow cytometry better predict rate of material transport across the BBB in mice than Transwell assays. Static treatment data is reported as mean ± standard deviation of three plate replicates. Flow chip “Uptake Under Flow” data display mean ± standard deviation for six technical replicate channels in series on one flow chip. Mouse BBB permeability data display mean ± standard deviation of each of four animals’ average measured permeability. Trendlines display simple linear regressions of the data, and dotted lines indicate the 90% confidence interval of the trendline.

| **Supplementary Table 1:** Gene expression values for transwell (Trans) and solid plasticware (Plastic) grown cells at given passage numbers (P#) and days of development (D#). All values are arithmetic mean of 3-4 well replicates. | | | | | | | | | | | | | | | | | | |
| --- | --- | --- | --- | --- | --- | --- | --- | --- | --- | --- | --- | --- | --- | --- | --- | --- | --- | --- |
|  | P2D4 | | P2D7 | | P2D14 | | P6D4 | | P6D7 | | P6D14 | | P10D4 | | P10D7 | | P10D14 | |
|  | Trans | Plastic | Trans | Plastic | Trans | Plastic | Trans | Plastic | Trans | Plastic | Trans | Plastic | Trans | Plastic | Trans | Plastic | Trans | Plastic |
| ACTB | 1.000 | 1.000 | 1.000 | 1.000 | 1.000 | 1.000 | 1.000 | 1.000 | 1.000 | 1.000 | 1.000 | 1.000 | 1.000 | 1.000 | 1.000 | 1.000 | 1.000 | 1.000 |
| TFRC | 0.021 | 0.020 | 0.092 | 0.100 | 0.034 | 0.041 | 0.063 | 0.083 | 0.022 | 0.024 | 0.017 | 0.017 | 0.024 | 0.037 | 0.057 | 0.045 | 0.015 | 0.016 |
| LRP1 | 0.048 | 0.037 | 0.034 | 0.044 | 0.077 | 0.065 | 0.025 | 0.027 | 0.027 | 0.018 | 0.042 | 0.035 | 0.020 | 0.012 | 0.030 | 0.017 | 0.048 | 0.041 |
| TJP1 | 0.233 | 0.155 | 0.103 | 0.180 | 0.235 | 0.291 | 0.049 | 0.058 | 0.113 | 0.074 | 0.111 | 0.118 | 0.154 | 0.088 | 0.239 | 0.242 | 0.165 | 0.133 |
| CDH5 | 0.026 | 0.013 | 0.050 | 0.045 | 0.035 | 0.037 | 0.030 | 0.028 | 0.023 | 0.026 | 0.018 | 0.011 | 0.008 | 0.006 | 0.027 | 0.030 | 0.009 | 0.007 |
| CD34 | 0.084 | 0.053 | 0.018 | 0.022 | 0.048 | 0.037 | 0.021 | 0.015 | 0.024 | 0.019 | 0.029 | 0.030 | 0.016 | 0.008 | 0.025 | 0.029 | 0.020 | 0.016 |
| VWF | 0.014 | 0.010 | 0.012 | 0.024 | 0.023 | 0.020 | 0.007 | 0.006 | 0.006 | 0.004 | 0.003 | 0.003 | 0.005 | 0.003 | 0.009 | 0.007 | 0.003 | 0.002 |
| PECAM1 | 0.049 | 0.034 | 0.012 | 0.012 | 0.107 | 0.055 | 0.009 | 0.008 | 0.020 | 0.011 | 0.018 | 0.014 | 0.014 | 0.007 | 0.019 | 0.025 | 0.014 | 0.022 |

| **Supplementary Table 2:** Nanoparticle characteristics for auxiliary nanoparticle batches, measured by dynamic light scattering. All values are displayed as mean ± SD of three runs.  PDI: polydispersity index. | | | |
| --- | --- | --- | --- |
| Nanoparticle ID | Number Average Size (nm) | PDI | Zeta Potential (mV) |
| Cy3-labelled Nanoparticles for *in vitro* confocal imaging | | | |
| Bare Lipo | 74.5 ± 2.6 | 0.09 ± 0.00 | -46.7 ± 2.8 |
| Lipo-PLR-HA | 87.0 ± 6.3 | 0.14 ± 0.03 | -33.5 ± 0.7 |
| Lipo-PLR-PLE | 84.1 ± 2.8 | 0.15 ± 0.02 | -54.1 ± 1.9 |
| Cy5-labelled nanoparticles for intravital imaging in mice | | | |
| Bare Lipo | 70.8 ± 4.0 | 0.15 ± 0.04 | -42.0 ± 2.1 |
| Lipo-PLR-CMDex | 102.1 ± 13.8 | 0.23 ± 0.09 | -34.8 ± 1.0 |
| Lipo-PLR-HA | 105.7 ± 12.5 | 0.16 ± 0.04 | -31.2 ± 0.5 |
| Lipo-PLR-PLD | 102.2 ± 12.2 | 0.15 ± 0.00 | -39.9 ±1.9 |
| Lipo-PLR-PLE | 106.9 ± 3.9 | 0.17 ± 0.06 | -44.3 ± 3.1 |
| Lipo-PLR-PSialA | 107.0 ± 7.7 | 0.05 ± 0.04 | -38.3 ± 0.9 |
| PLGA-PLR-PLD | 134.4 ± 9.2 | 0.22 ± 0.05 | -43.0 ± 1.0 |
| PS-PLR-PLD | 132.4 ± 2.7 | 0.22 ± 0.09 | -46.4 ± 2.17 |

| **Supplementary Table 3: Tabulated blood brain barrier permeability values for intravital imaging, displaying nanoparticle permeability (P NP), dextran permeability P FD70, and the ratio thereof for each animal.** | | | | | | | | | | | | | | | |
| --- | --- | --- | --- | --- | --- | --- | --- | --- | --- | --- | --- | --- | --- | --- | --- |
|  | Imaging Session 1 | | | | | Imaging Session 2 | | | | | Imaging Session 3 | | | | |
| ID | P NP (μm/s) | P NP (cm/s) | P FD70(μm/s) | P FD70(cm/s) | P[NP]/P[FD70] | P NP (μm/s) | P NP (cm/s) | P FD70(μm/s) | P FD70(cm/s) | P[NP]/P[FD70] | P NP (μm/s) | P NP (cm/s) | P FD70(μm/s) | P FD70(cm/s) | P[NP]/P[FD70] |
|  |  |  |  |  |  |  |  |  |  |  |  |  |  |  |  |
| PLD-1-F | 0.00124 | 1.25E-07 | 0.00374 | 3.75E-07 | 0.33247 | 0.00269 | 2.7E-07 | 0.00208 | 2.08E-07 | 0.7198 | Image set cannot be processed | | | | |
|  | 0.00107 | 1.07E-07 | 0.00184 | 1.85E-07 | 0.57892 | 0.00332 | 3.32E-07 | 0.00110 | 1.1E-07 | 1.7981 |  |  |  |  |  |
|  | 0.00328 | 3.28E-07 | 0.00144 | 1.45E-07 | 2.27158 | Remainder of image set cannot be processed | | | | |  |  |  |  |  |
|  | 0.00092 | 9.21E-08 | 0.00093 | 9.3E-08 | 0.99062 |  |  |  |  |  |  |  |  |  |  |
|  | 0.00208 | 2.08E-07 | 0.00087 | 8.78E-08 | 2.37299 |  |  |  |  |  |  |  |  |  |  |
|  |  |  |  |  |  |  |  |  |  |  |  |  |  |  |  |
| PLD-2-F | Image set cannot be processed | | | | | 0.00211 | 2.11E-07 | 0.00142 | 1.42E-07 | 1.485462 | Image set cannot be processed | | | | |
|  |  |  |  |  |  | 0.002316 | 2.32E-07 | 0.000684 | 6.84E-08 | 3.38697 |  |  |  |  |  |
|  |  |  |  |  |  | 0.000382 | 3.82E-08 | 0.000748 | 7.48E-08 | 0.510878 |  |  |  |  |  |
|  |  |  |  |  |  | 0.000885 | 8.85E-08 | 0.00067 | 6.7E-08 | 1.319954 |  |  |  |  |  |
|  |  |  |  |  |  | 0.002046 | 2.05E-07 | 0.000707 | 7.07E-08 | 2.892494 |  |  |  |  |  |
|  |  |  |  |  |  |  |  |  |  |  |  |  |  |  |  |
| PLD-3-M | 0.010244 | 1.02E-06 | 0.005477 | 5.48E-07 | 1.870354 | Image set cannot be processed | | | | | Image set cannot be processed | | | | |
|  | 0.003667 | 3.67E-07 | 0.002431 | 2.43E-07 | 1.50879 |  |  |  |  |  |  |  |  |  |  |
|  | 0.003999 | 4E-07 | 0.001611 | 1.61E-07 | 2.483061 |  |  |  |  |  |  |  |  |  |  |
|  | 0.002591 | 2.59E-07 | 0.001289 | 1.29E-07 | 2.010978 |  |  |  |  |  |  |  |  |  |  |
|  | 0.000777 | 7.77E-08 | 0.000988 | 9.88E-08 | 0.786134 |  |  |  |  |  |  |  |  |  |  |
|  |  |  |  |  |  |  |  |  |  |  |  |  |  |  |  |
| PLD-4-M | 0.002987 | 2.99E-07 | 0.002793 | 2.79E-07 | 1.069442 | Image set cannot be processed | | | | | 0.012967 | 1.3E-06 | 0.004379 | 4.38E-07 | 2.960979 |
|  | 0.001381 | 1.38E-07 | 0.001448 | 1.45E-07 | 0.953902 |  |  |  |  |  | 0.005447 | 5.45E-07 | 0.002185 | 2.18E-07 | 2.493203 |
|  | 0.000444 | 4.44E-08 | 0.000584 | 5.84E-08 | 0.761024 |  |  |  |  |  | 0.003621 | 3.62E-07 | 0.001801 | 1.8E-07 | 2.011084 |
|  | 0.000323 | 3.23E-08 | 0.00048 | 4.8E-08 | 0.673027 |  |  |  |  |  | 0.00092 | 9.2E-08 | 0.001465 | 1.47E-07 | 0.62789 |
|  | 0.000355 | 3.55E-08 | 0.000523 | 5.23E-08 | 0.679131 |  |  |  |  |  | 0.001214 | 1.21E-07 | 0.001111 | 1.11E-07 | 1.093149 |
|  |  |  |  |  |  |  |  |  |  |  |  |  |  |  |  |
|  |  |  |  |  |  |  |  |  |  |  |  |  |  |  |  |
| PLGA-1-F | 0.001731 | 1.73E-07 | 0.000833 | 8.33E-08 | 2.078071 | 0.001901 | 1.9E-07 | 0.003754 | 3.75E-07 | 0.506234 | 0.002147 | 2.15E-07 | 0.003911 | 3.91E-07 | 0.549053 |
|  | 0.000805 | 8.05E-08 | 0.000376 | 3.76E-08 | 2.141985 | 0.002091 | 2.09E-07 | 0.001894 | 1.89E-07 | 1.104329 | 0.002755 | 2.75E-07 | 0.00215 | 2.15E-07 | 1.281032 |
|  | 0.000504 | 5.04E-08 | 0.000569 | 5.69E-08 | 0.886147 | 0.002451 | 2.45E-07 | 0.002029 | 2.03E-07 | 1.207595 | 0.002467 | 2.47E-07 | 0.001465 | 1.47E-07 | 1.683735 |
|  | 0.000491 | 4.91E-08 | 0.000749 | 7.49E-08 | 0.655534 | Image set cannot be processed | | | | | 0.001898 | 1.9E-07 | 0.001295 | 1.29E-07 | 1.466158 |
|  | 0.000362 | 3.62E-08 | 0.000988 | 9.88E-08 | 0.366882 |  |  |  |  |  | 0.001628 | 1.63E-07 | 0.001232 | 1.23E-07 | 1.321782 |
|  |  |  |  |  |  |  |  |  |  |  |  |  |  |  |  |
| PLGA-2-F | 0.001435 | 1.44E-07 | 0.001681 | 1.68E-07 | 0.85383 | 0.001216 | 1.22E-07 | 0.004821 | 4.82E-07 | 0.252323 | Image set cannot be processed | | | | |
|  | 0.001346 | 1.35E-07 | 0.001669 | 1.67E-07 | 0.806342 | 0.003639 | 3.64E-07 | 0.00219 | 2.19E-07 | 1.661437 |  |  |  |  |  |
|  | 0.001037 | 1.04E-07 | 0.001543 | 1.54E-07 | 0.672077 | 0.002412 | 2.41E-07 | 0.001822 | 1.82E-07 | 1.323429 |  |  |  |  |  |
|  | 0.001063 | 1.06E-07 | 0.001368 | 1.37E-07 | 0.776882 | 0.001914 | 1.91E-07 | 0.00143 | 1.43E-07 | 1.337773 |  |  |  |  |  |
|  | 0.000929 | 9.29E-08 | 0.001252 | 1.25E-07 | 0.741777 | 0.001413 | 1.41E-07 | 0.001177 | 1.18E-07 | 1.201321 |  |  |  |  |  |
|  |  |  |  |  |  |  |  |  |  |  |  |  |  |  |  |
| PLGA-3-M | Image set cannot be processed | | | | | -0.00981 | -9.8E-07 | 0.000643 | 6.43E-08 | -15.2591 | 0.000711 | 7.11E-08 | 0.002011 | 2.01E-07 | 0.353701 |
|  |  |  |  |  |  | -0.00238 | -2.4E-07 | 0.000603 | 6.03E-08 | -3.94157 | -4.4E-05 | -4.4E-09 | 0.002024 | 2.02E-07 | -0.02166 |
|  |  |  |  |  |  | -0.00204 | -2E-07 | 0.000364 | 3.64E-08 | -5.59815 | -0.00131 | -1.3E-07 | 0.002011 | 2.01E-07 | -0.65267 |
|  |  |  |  |  |  | 0.000801 | 8.01E-08 | 0.000711 | 7.11E-08 | 1.126644 | -0.00148 | -1.5E-07 | 0.00184 | 1.84E-07 | -0.8051 |
|  |  |  |  |  |  | 0.002103 | 2.1E-07 | 0.000701 | 7.01E-08 | 3.000126 | -0.00086 | -8.6E-08 | 0.001727 | 1.73E-07 | -0.49664 |
|  |  |  |  |  |  |  |  |  |  |  |  |  |  |  |  |
| PLGA-4-M | 0.003457 | 3.46E-07 | 0.003238 | 3.24E-07 | 1.067662 | 0.002132 | 2.13E-07 | 0.002498 | 2.5E-07 | 0.853389 | -0.00146 | -1.5E-07 | 0.002346 | 2.35E-07 | -0.62034 |
|  | 0.003322 | 3.32E-07 | 0.002345 | 2.35E-07 | 1.416523 | 0.001972 | 1.97E-07 | 0.00228 | 2.28E-07 | 0.864923 | -0.00015 | -1.5E-08 | 0.001299 | 1.3E-07 | -0.11893 |
|  | 0.000892 | 8.92E-08 | 0.00229 | 2.29E-07 | 0.389571 | 0.002612 | 2.61E-07 | 0.001404 | 1.4E-07 | 1.859861 | 4.29E-05 | 4.29E-09 | 0.001138 | 1.14E-07 | 0.037718 |
|  | 0.001086 | 1.09E-07 | 0.001173 | 1.17E-07 | 0.926188 | 0.002319 | 2.32E-07 | 0.001273 | 1.27E-07 | 1.821442 | 0.000322 | 3.22E-08 | 0.000853 | 8.53E-08 | 0.377532 |
|  | 0.001684 | 1.68E-07 | 0.001351 | 1.35E-07 | 1.246388 | 0.002484 | 2.48E-07 | 0.001009 | 1.01E-07 | 2.461049 | 0.000407 | 4.07E-08 | 0.000828 | 8.28E-08 | 0.491648 |
|  |  |  |  |  |  |  |  |  |  |  |  |  |  |  |  |
| PS-1-F | 0.001524 | 1.52E-07 | 0.001285 | 1.29E-07 | 1.185468 | 0.001767 | 1.77E-07 | 0.001934 | 1.93E-07 | 0.913551 | 0.001399 | 1.4E-07 | 0.000983 | 9.83E-08 | 1.422089 |
|  | 0.001631 | 1.63E-07 | 0.001515 | 1.51E-07 | 1.076333 | 0.001001 | 1E-07 | 0.001084 | 1.08E-07 | 0.922811 | 0.000835 | 8.35E-08 | 0.000634 | 6.34E-08 | 1.316441 |
|  | 0.001509 | 1.51E-07 | 0.001468 | 1.47E-07 | 1.027684 | 0.001185 | 1.18E-07 | 0.001309 | 1.31E-07 | 0.905305 | 0.000906 | 9.06E-08 | 0.000754 | 7.54E-08 | 1.202447 |
|  | 0.000927 | 9.27E-08 | 0.00097 | 9.7E-08 | 0.955245 | 0.001347 | 1.35E-07 | 0.001378 | 1.38E-07 | 0.977586 | 0.000859 | 8.59E-08 | 0.00074 | 7.4E-08 | 1.160874 |
|  | 0.000616 | 6.16E-08 | 0.000848 | 8.48E-08 | 0.726419 | 0.001236 | 1.24E-07 | 0.001031 | 1.03E-07 | 1.19886 | 0.000801 | 8.01E-08 | 0.0006 | 6E-08 | 1.334365 |
|  |  |  |  |  |  |  |  |  |  |  |  |  |  |  |  |
| PS-2-F | 0.001837 | 1.84E-07 | 0.002099 | 2.1E-07 | 0.875012 | Image set cannot be processed | | | | | Image set cannot be processed | | | | |
|  | 0.001417 | 1.42E-07 | 0.00145 | 1.45E-07 | 0.977494 |  |  |  |  |  |  |  |  |  |  |
|  | 0.001278 | 1.28E-07 | 0.001105 | 1.11E-07 | 1.156831 |  |  |  |  |  |  |  |  |  |  |
|  | 0.000876 | 8.76E-08 | 0.000847 | 8.47E-08 | 1.03446 |  |  |  |  |  |  |  |  |  |  |
|  | 0.000842 | 8.42E-08 | 0.000653 | 6.53E-08 | 1.288002 |  |  |  |  |  |  |  |  |  |  |
|  |  |  |  |  |  |  |  |  |  |  |  |  |  |  |  |
| PS-3-M | 0.001206 | 1.21E-07 | 0.001926 | 1.93E-07 | 0.62648 | Image set cannot be processed | | | | | Image set cannot be processed | | | | |
|  | 0.000987 | 9.87E-08 | 0.001485 | 1.49E-07 | 0.664753 |  |  |  |  |  |  |  |  |  |  |
|  | 0.000808 | 8.08E-08 | 0.001329 | 1.33E-07 | 0.607577 |  |  |  |  |  |  |  |  |  |  |
|  | Remainder of image set cannot be processed | | | | |  |  |  |  |  |  |  |  |  |  |
|  |  |  |  |  |  |  |  |  |  |  |  |  |  |  |  |
| PS-4-M | 0.002437 | 2.44E-07 | 0.002852 | 2.85E-07 | 0.854347 | 0.003046 | 3.05E-07 | 0.002975 | 2.97E-07 | 1.023991 | 0.003762 | 3.76E-07 | 0.00386 | 3.86E-07 | 0.974422 |
|  | 0.001509 | 1.51E-07 | 0.001798 | 1.8E-07 | 0.839588 | 0.001988 | 1.99E-07 | 0.002072 | 2.07E-07 | 0.959197 | 0.001487 | 1.49E-07 | 0.001481 | 1.48E-07 | 1.003829 |
|  | 0.001158 | 1.16E-07 | 0.001451 | 1.45E-07 | 0.798077 | 0.001856 | 1.86E-07 | 0.001873 | 1.87E-07 | 0.990523 | 0.001672 | 1.67E-07 | 0.001651 | 1.65E-07 | 1.012825 |
|  | 0.001117 | 1.12E-07 | 0.001437 | 1.44E-07 | 0.776998 | 0.000465 | 4.65E-08 | 0.000513 | 5.13E-08 | 0.906239 | 0.000731 | 7.31E-08 | 0.000736 | 7.36E-08 | 0.99271 |
|  | 0.000983 | 9.83E-08 | 0.001247 | 1.25E-07 | 0.787928 | 0.000465 | 4.65E-08 | 0.000488 | 4.88E-08 | 0.952613 | 0.000575 | 5.75E-08 | 0.00062 | 6.2E-08 | 0.92752 |
|  |  |  |  |  |  |  |  |  |  |  |  |  |  |  |  |
| Lipo-1-F | 0.003998 | 4E-07 | 0.001621 | 1.62E-07 | 2.466389 | Image set cannot be processed | | | | | Image set cannot be processed | | | | |
|  | 0.001621 | 1.62E-07 | 0.000869 | 8.69E-08 | 1.864439 |  |  |  |  |  |  |  |  |  |  |
|  | 0.000246 | 2.46E-08 | 0.000288 | 2.88E-08 | 0.855538 |  |  |  |  |  |  |  |  |  |  |
|  | Remainder of image set cannot be processed | | | | |  |  |  |  |  |  |  |  |  |  |
|  |  |  |  |  |  |  |  |  |  |  |  |  |  |  |  |
|  |  |  |  |  |  |  |  |  |  |  |  |  |  |  |  |
| Lipo-2-F | 0.000584 | 5.84E-08 | 0.001026 | 1.03E-07 | 0.569369 | 0.007149 | 7.15E-07 | 0.005471 | 5.47E-07 | 1.306752 | 0.002053 | 2.05E-07 | 0.004391 | 4.39E-07 | 0.467682 |
|  | 0.000352 | 3.52E-08 | 0.000743 | 7.43E-08 | 0.473299 | 0.006036 | 6.04E-07 | 0.003157 | 3.16E-07 | 1.912144 | 0.002825 | 2.82E-07 | 0.002436 | 2.44E-07 | 1.159596 |
|  | Remainder of image set cannot be processed | | | | | 0.005019 | 5.02E-07 | 0.002532 | 2.53E-07 | 1.982065 | 0.003304 | 3.3E-07 | 0.001466 | 1.47E-07 | 2.253825 |
|  |  |  |  |  |  | 0.00108 | 1.08E-07 | 0.000994 | 9.94E-08 | 1.086983 | 0.002985 | 2.99E-07 | 0.001095 | 1.09E-07 | 2.727403 |
|  |  |  |  |  |  | 0.002277 | 2.28E-07 | 0.001444 | 1.44E-07 | 1.57637 | 0.001638 | 1.64E-07 | 0.000936 | 9.36E-08 | 1.750321 |
|  |  |  |  |  |  |  |  |  |  |  |  |  |  |  |  |
| Lipo-3-M | 0.002661 | 2.66E-07 | 0.00209 | 2.09E-07 | 1.273161 | 0.002549 | 2.55E-07 | 0.00192 | 1.92E-07 | 1.327633 | Image set cannot be processed | | | | |
|  | 0.001304 | 1.3E-07 | 0.001157 | 1.16E-07 | 1.12769 | 0.001324 | 1.32E-07 | 0.001438 | 1.44E-07 | 0.920257 |  |  |  |  |  |
|  | Remainder of image set cannot be processed | | | | | 0.00073 | 7.3E-08 | 0.001137 | 1.14E-07 | 0.642113 |  |  |  |  |  |
|  |  |  |  |  |  | 0.000304 | 3.04E-08 | 0.000722 | 7.22E-08 | 0.42044 |  |  |  |  |  |
|  |  |  |  |  |  | 0.000245 | 2.45E-08 | 0.000539 | 5.39E-08 | 0.454673 |  |  |  |  |  |
|  |  |  |  |  |  |  |  |  |  |  |  |  |  |  |  |
| Lipo-4-M | Image set cannot be processed | | | | | Image set cannot be processed | | | | | 0.004239 | 4.24E-07 | 0.004764 | 4.76E-07 | 0.889799 |
|  |  |  |  |  |  |  |  |  |  |  | 0.011428 | 1.14E-06 | 0.00295 | 2.95E-07 | 3.874331 |
|  |  |  |  |  |  |  |  |  |  |  | 0.003042 | 3.04E-07 | 0.002015 | 2.02E-07 | 1.509258 |
|  |  |  |  |  |  |  |  |  |  |  | 0.002155 | 2.15E-07 | 0.001244 | 1.24E-07 | 1.731322 |
|  |  |  |  |  |  |  |  |  |  |  | 0.001067 | 1.07E-07 | 0.000921 | 9.21E-08 | 1.159555 |
| CMDex-1-F | 0.001625 | 1.63E-07 | 0.001728 | 1.73E-07 | 1.588129 | 0.003029 | 3.03E-07 | 0.001023 | 1.02E-07 | 2.959266 | 0.000145 | 1.45E-08 | 0.001512 | 1.51E-07 | 0.096026 |
|  | 0.000768 | 7.68E-08 | 0.001103 | 1.1E-07 | 1.293249 | 0.001483 | 1.48E-07 | 0.000593 | 5.93E-08 | 2.498143 | 0.001574 | 1.57E-07 | 0.00103 | 1.03E-07 | 1.527812 |
|  | 0.00069 | 6.9E-08 | 0.000596 | 5.96E-08 | 2.127534 | 0.000431 | 4.31E-08 | 0.000324 | 3.24E-08 | 1.32868 | 0.000537 | 5.37E-08 | 0.000581 | 5.81E-08 | 0.923255 |
|  | 0.000374 | 3.74E-08 | 0.000487 | 4.87E-08 | 1.358822 | 0.00054 | 5.4E-08 | 0.000275 | 2.75E-08 | 1.960814 | 0.000447 | 4.47E-08 | 0.000417 | 4.17E-08 | 1.070759 |
|  | 0.00029 | 2.9E-08 | 0.000342 | 3.42E-08 | 0.911139 | 0.000431 | 4.31E-08 | 0.000319 | 3.19E-08 | 1.352362 | 0.00072 | 7.2E-08 | 0.000359 | 3.59E-08 | 2.004221 |
|  |  |  |  |  |  |  |  |  |  |  |  |  |  |  |  |
| CMDex-2-F | Image set cannot be processed | | | | | 0.001424 | 1.42E-07 | 0.001923 | 1.92E-07 | 0.740698 | 0.001803 | 1.8E-07 | 0.001343 | 1.34E-07 | 1.342652 |
|  |  |  |  |  |  | 0.001619 | 1.62E-07 | 0.001469 | 1.47E-07 | 1.101904 | 0.002167 | 2.17E-07 | 0.0009 | 9E-08 | 2.40946 |
|  |  |  |  |  |  | 0.000977 | 9.77E-08 | 0.000853 | 8.53E-08 | 1.145324 | 0.002334 | 2.33E-07 | 0.000752 | 7.52E-08 | 3.103163 |
|  |  |  |  |  |  | 0.002623 | 2.62E-07 | 0.001044 | 1.04E-07 | 2.513223 | 0.001992 | 1.99E-07 | 0.000864 | 8.64E-08 | 2.304491 |
|  |  |  |  |  |  | 0.001685 | 1.68E-07 | 0.000811 | 8.11E-08 | 2.076211 | Remainder of image set cannot be processed | | | | |
|  |  |  |  |  |  |  |  |  |  |  |  |  |  |  |  |
| CMDex-3-M | Image set cannot be processed | | | | | 0.003071 | 3.07E-07 | 0.003062 | 3.06E-07 | 1.002869 | 0.00197 | 1.97E-07 | 0.000924 | 9.24E-08 | 2.133058 |
|  |  |  |  |  |  | 0.001855 | 1.85E-07 | 0.002046 | 2.05E-07 | 0.906444 | 0.000341 | 3.41E-08 | 0.000713 | 7.13E-08 | 0.478724 |
|  |  |  |  |  |  | 0.001362 | 1.36E-07 | 0.001273 | 1.27E-07 | 1.06979 | 0.000205 | 2.05E-08 | 0.000749 | 7.49E-08 | 0.273856 |
|  |  |  |  |  |  | 0.001065 | 1.06E-07 | 0.00109 | 1.09E-07 | 0.976601 | 0.000577 | 5.77E-08 | 0.000835 | 8.35E-08 | 0.690703 |
|  |  |  |  |  |  | 0.001079 | 1.08E-07 | 0.000996 | 9.96E-08 | 1.083073 | 0.001199 | 1.2E-07 | 0.000723 | 7.23E-08 | 1.659689 |
|  |  |  |  |  |  |  |  |  |  |  |  |  |  |  |  |
| CMDex-4-M | 0.011814 | 1.18E-06 | 0.005319 | 5.32E-07 | 2.221015 | 0.000534 | 5.34E-08 | 0.00095 | 9.5E-08 | 0.562104 | Did not take a third set of images | | | | |
|  | 0.008025 | 8.03E-07 | 0.002826 | 2.83E-07 | 2.840333 | 0.00127 | 1.27E-07 | 0.000733 | 7.33E-08 | 1.732934 |  |  |  |  |  |
|  | Image set cannot be processed | | | | | Image set cannot be processed | | | | |  |  |  |  |  |
|  |  |  |  |  |  |  |  |  |  |  |  |  |  |  |  |
|  |  |  |  |  |  |  |  |  |  |  |  |  |  |  |  |
| HA-1-F | 0.009125 | 9.12E-07 | 0.004715 | 4.71E-07 | 1.93535 | 0.005243 | 5.24E-07 | 0.003196 | 3.2E-07 | 1.640371 | Image set cannot be processed | | | | |
|  | 0.003315 | 3.31E-07 | 0.001609 | 1.61E-07 | 2.060158 | 0.003087 | 3.09E-07 | 0.00131 | 1.31E-07 | 2.356154 |  |  |  |  |  |
|  | 0.001776 | 1.78E-07 | 0.001357 | 1.36E-07 | 1.309188 | Remainder of image set cannot be processed | | | | |  |  |  |  |  |
|  | 0.003304 | 3.3E-07 | 0.00131 | 1.31E-07 | 2.52181 |  |  |  |  |  |  |  |  |  |  |
|  | 0.00243 | 2.43E-07 | 0.001146 | 1.15E-07 | 2.121033 |  |  |  |  |  |  |  |  |  |  |
|  |  |  |  |  |  |  |  |  |  |  |  |  |  |  |  |
| HA-2-F | 0.027723 | 2.77E-06 | 0.006559 | 3 | 4.226755 | 0.004603 | 4.6E-07 | 0.00276 | 4 | 1.668102 | 0.00796 | 7.96E-07 | 0.002773 | 2.77E-07 | 2.870703 |
|  | 0.016848 | 1.68E-06 | 0.003383 | 3.38E-07 | 4.980654 | 0.003271 | 3.27E-07 | 0.001435 | 1.44E-07 | 2.278967 | 0.004721 | 4.72E-07 | 0.001824 | 1.82E-07 | 2.588488 |
|  | 0.014754 | 1.48E-06 | 0.002286 | 2.29E-07 | 6.454282 | Remainder of image set cannot be processed | | | | | 0.001909 | 1.91E-07 | 0.001272 | 1.27E-07 | 1.501005 |
|  | 0.010219 | 1.02E-06 |  | 2.11E-07 | 4.834379 |  |  |  |  |  | 0.001748 | 1.75E-07 | 0.000896 | 8.96E-08 | 1.950009 |
|  | 0.009634 | 9.63E-07 | 0.001896 | 1.9E-07 | 5.082189 |  |  |  |  |  | 0.001358 | 1.36E-07 | 0.000753 | 7.53E-08 | 1.804411 |
| HA-3-M | Issues with Z shift; ignore this time point | | | | | 0.003519 | 3.52E-07 | 0.000686 | 6.86E-08 | 5.129614 | 0.001485 | 1.49E-07 | 0.001077 | 1.08E-07 | 1.378944 |
|  | 0.004882 | 4.88E-07 | 0.001323 | 1.32E-07 | 3.689827 | 0.005773 | 5.77E-07 | 0.003371 | 3.37E-07 | 1.712602 | 0.002941 | 2.94E-07 | 0.000931 | 9.31E-08 | 3.160244 |
|  | 0.003939 | 3.94E-07 | 0.001034 | 1.03E-07 | 3.809929 | Remainder of image set cannot be processed | | | | | Remainder of image set cannot be processed | | | | |
|  | 0.003161 | 3.16E-07 | 0.000748 | 7.48E-08 | 4.22639 |  |  |  |  |  |  |  |  |  |  |
|  | 0.002176 | 2.18E-07 | 0.000585 | 5.85E-08 | 3.718212 |  |  |  |  |  |  |  |  |  |  |
|  |  |  |  |  |  |  |  |  |  |  |  |  |  |  |  |
| HA-4-M | 0.000149 | 1.49E-08 | 0.002712 | 2.71E-07 | 0.055118 | 0.006223 | 6.22E-07 | 0.001959 | 1.96E-07 | 2.300723 | 0.004397 | 4.4E-07 | 0.001735 | 1.73E-07 | 2.534353 |
|  | 0.001058 | 1.06E-07 | 0.001769 | 1.77E-07 | 0.598076 | 0.003944 | 3.94E-07 | 0.001062 | 1.06E-07 | 3.713065 | 0.002142 | 2.14E-07 | 0.001171 | 1.17E-07 | 1.828217 |
|  | 0.002748 | 2.75E-07 | 0.001075 | 1.08E-07 | 2.556116 | 0.003341 | 3.34E-07 | 0.000902 | 9.02E-08 | 5.116901 | 0.001426 | 1.43E-07 | 0.000791 | 7.91E-08 | 1.801645 |
|  | 0.003106 | 3.11E-07 | 0.001021 | 1.02E-07 | 3.043301 | 0.003244 | 3.24E-07 | 0.000846 | 8.46E-08 | 3.835477 | 0.000448 | 4.48E-08 | 0.00076 | 7.6E-08 | 0.589774 |
|  | Remainder of image set cannot be processed | | | | | 0.002795 | 2.79E-07 | 0.000899 | 8.99E-08 | 3.109119 | 0.000276 | 2.76E-08 | 0.000365 | 3.65E-08 | 0.757095 |
|  |  |  |  |  |  |  |  |  |  |  |  |  |  |  |  |
| PLE-1-F | 0.112797 | 1.13E-05 | 0.11436 | 1.14E-05 | 0.986334 | Image set cannot be processed | | | | | 0.015689 | 1.57E-06 | 0.02341 | 2.34E-06 | 0.670172 |
|  | 0.083463 | 8.35E-06 | 0.108453 | 1.08E-05 | 0.769572 |  |  |  |  |  | 0.006709 | 6.71E-07 | 0.010892 | 1.09E-06 | 0.616002 |
|  | 0.085827 | 8.58E-06 | 0.101337 | 1.01E-05 | 0.846949 |  |  |  |  |  | 0.007693 | 7.69E-07 | 0.008904 | 8.9E-07 | 0.863924 |
|  | 0.072809 | 7.28E-06 | 0.079629 | 7.96E-06 | 0.914357 |  |  |  |  |  | 0.006551 | 6.55E-07 | 0.005972 | 5.97E-07 | 1.097001 |
|  | 0.075425 | 7.54E-06 | 0.078624 | 7.86E-06 | 0.959316 |  |  |  |  |  | 0.004527 | 4.53E-07 | 0.005356 | 5.36E-07 | 0.845242 |
|  |  |  |  |  |  |  |  |  |  |  |  |  |  |  |  |
| PLE-2-F | Image set cannot be processed | | | | | Image set cannot be processed | | | | | 0.003812 | 3.81E-07 | 0.003672 | 3.67E-07 | 1.038204 |
|  |  |  |  |  |  |  |  |  |  |  | 0.003169 | 3.17E-07 | 0.001521 | 1.52E-07 | 2.08418 |
|  |  |  |  |  |  |  |  |  |  |  | 0.001522 | 1.52E-07 | 0.00081 | 8.1E-08 | 1.878528 |
|  |  |  |  |  |  |  |  |  |  |  | 0.002288 | 2.29E-07 | 0.000628 | 6.28E-08 | 3.644751 |
|  |  |  |  |  |  |  |  |  |  |  | 0.000626 | 6.26E-08 | 0.000583 | 5.83E-08 | 1.074291 |
|  |  |  |  |  |  |  |  |  |  |  |  |  |  |  |  |
| PLE-3-M | Image set cannot be processed | | | | | 0.004102 | 4.1E-07 | 0.002638 | 2.64E-07 | 1.555058 | Image set cannot be processed | | | | |
|  |  |  |  |  |  | 0.00071 | 7.1E-08 | 0.001316 | 1.32E-07 | 0.53901 |  |  |  |  |  |
|  |  |  |  |  |  | 0.001262 | 1.26E-07 | 0.001067 | 1.07E-07 | 1.181965 |  |  |  |  |  |
|  |  |  |  |  |  | Image set cannot be processed | | | | |  |  |  |  |  |
|  |  |  |  |  |  |  |  |  |  |  |  |  |  |  |  |
| PLE-4-M | Image set cannot be processed | | | | | 0.000575 | 5.75E-08 | 0.000233 | 2.33E-08 | 2.465424 | Image set cannot be processed | | | | |
|  |  |  |  |  |  | 0.000694 | 6.94E-08 | 0.000279 | 2.79E-08 | 0.385978 |  |  |  |  |  |
|  |  |  |  |  |  | 0.000301 | 3.01E-08 | 0.00034 | 3.4E-08 | 0.207199 |  |  |  |  |  |
|  |  |  |  |  |  | Remainder of image set cannot be processed | | | | |  |  |  |  |  |
|  |  |  |  |  |  |  |  |  |  |  |  |  |  |  |  |
| PLE.TV-1-F | 0.00085 | 8.5E-08 | 0.001536 | 1.54E-07 | 0.553114 | Image set cannot be processed | | | | | Image set cannot be processed | | | | |
|  | 0.00197 | 1.97E-07 | 0.001798 | 1.8E-07 | 1.095182 |  |  |  |  |  |  |  |  |  |  |
|  | 0.002383 | 2.38E-07 | 0.001363 | 1.36E-07 | 1.74824 |  |  |  |  |  |  |  |  |  |  |
|  | 0.000353 | 3.53E-08 | 0.000647 | 6.47E-08 | 0.545564 |  |  |  |  |  |  |  |  |  |  |
|  | 0.001582 | 1.58E-07 | 0.000751 | 7.51E-08 | 2.107403 |  |  |  |  |  |  |  |  |  |  |
|  |  |  |  |  |  |  |  |  |  |  |  |  |  |  |  |
| PLE.TV-2-F | Image set cannot be processed | | | | | 0.001095 | 1.1E-07 | 0.001119 | 1.12E-07 | 0.979227 | 0.003336 | 3.34E-07 | 0.001214 | 1.21E-07 | 2.748422 |
|  |  |  |  |  |  | 0.00111 | 1.11E-07 | 0.00065 | 6.5E-08 | 1.707672 | 0.000221 | 2.21E-08 | 0.000598 | 5.98E-08 | 0.369447 |
|  |  |  |  |  |  | 0.000722 | 7.22E-08 | 0.000335 | 3.35E-08 | 2.156233 | 0.000265 | 2.65E-08 | 0.000361 | 3.61E-08 | 0.73465 |
|  |  |  |  |  |  | 0.000665 | 6.65E-08 | 0.000287 | 2.87E-08 | 2.319262 | 0.000663 | 6.63E-08 | 0.000315 | 3.15E-08 | 2.107361 |
|  |  |  |  |  |  | 0.000529 | 5.29E-08 | 0.000289 | 2.89E-08 | 1.832143 | 0.000368 | 3.68E-08 | 0.000235 | 2.35E-08 | 1.5673 |
|  |  |  |  |  |  |  |  |  |  |  |  |  |  |  |  |
| PLE.TV-3-M | 0.010589 | 1.06E-06 | 0.011147 | 1.11E-06 | 0.949981 | 0.001522 | 1.52E-07 | 0.003206 | 3.21E-07 | 0.474601 | 0.011633 | 1.16E-06 | 0.003207 | 3.21E-07 | 3.627724 |
|  | 0.007524 | 7.52E-07 | 0.004601 | 4.6E-07 | 1.635186 | 0.002214 | 2.21E-07 | 0.001485 | 1.48E-07 | 1.491217 | 0.005311 | 5.31E-07 | 0.001638 | 1.64E-07 | 3.242532 |
|  | Remainder of image set cannot be processed | | | | | 0.001451 | 1.45E-07 | 0.000983 | 9.83E-08 | 1.47615 | 0.001302 | 1.3E-07 | 0.000685 | 6.85E-08 | 1.900885 |
|  |  |  |  |  |  | 0.001198 | 1.2E-07 | 0.000775 | 7.75E-08 | 1.546512 | 0.000349 | 3.49E-08 | 0.000517 | 5.17E-08 | 0.674182 |
|  |  |  |  |  |  | 0.000317 | 3.17E-08 | 0.000753 | 7.53E-08 | 0.420361 | 0.000321 | 3.21E-08 | 0.000434 | 4.34E-08 | 0.73868 |
|  |  |  |  |  |  |  |  |  |  |  |  |  |  |  |  |
| PLE.TV-4-M | Image set cannot be processed | | | | | Image set cannot be processed | | | | | 0.002622 | 2.62E-07 | 0.003243 | 3.24E-07 | 0.808577 |
|  |  |  |  |  |  |  |  |  |  |  | 0.004419 | 4.42E-07 | 0.001317 | 1.32E-07 | 3.354369 |
|  |  |  |  |  |  |  |  |  |  |  | 0.002496 | 2.5E-07 | 0.001233 | 1.23E-07 | 2.024146 |
|  |  |  |  |  |  |  |  |  |  |  | 0.00014 | 1.4E-08 | 0.00094 | 9.4E-08 | 0.148604 |
|  |  |  |  |  |  |  |  |  |  |  | Remainder of image set cannot be processed | | | | |
|  |  |  |  |  |  |  |  |  |  |  |  |  |  |  |  |
| PSialA-1-F | Image set cannot be processed | | | | | 0.005457 | 5.46E-07 | 0.002368 | 2.37E-07 | 2.3046 | Image set cannot be processed | | | | |
|  |  |  |  |  |  | 0.003557 | 3.56E-07 | 0.001399 | 1.4E-07 | 2.542275 |  |  |  |  |  |
|  |  |  |  |  |  | 0.002215 | 2.21E-07 | 0.000837 | 8.37E-08 | 2.646087 |  |  |  |  |  |
|  |  |  |  |  |  | 0.000962 | 3.02E-07 | 0.000687 | 6.87E-08 | 1.401626 |  |  |  |  |  |
|  |  |  |  |  |  | 0.001799 | 1.8E-07 | 0.000685 | 6.85E-08 | 2.626568 |  |  |  |  |  |
|  |  |  |  |  |  |  |  |  |  |  |  |  |  |  |  |
| PSialA-2-F | Image set cannot be processed | | | | | 0.004974 | 4.97E-07 | 0.002546 | 2.55E-07 | 1.953667 | Issues with Z shift; ignore these time points | | | | |
|  |  |  |  |  |  | 0.003132 | 3.13E-07 | 0.001418 | 1.42E-07 | 2.208291 |  |  |  |  |  |
|  |  |  |  |  |  | 0.002803 | 2.8E-07 | 0.000966 | 9.66E-08 | 2.900385 | 0.002371 | 2.37E-07 | 0.002475 | 2.47E-07 | 0.958063 |
|  |  |  |  |  |  | 0.002079 | 2.08E-07 | 0.000714 | 7.14E-08 | 2.913675 | 0.002202 | 2.2E-07 | 0.002584 | 2.58E-07 | 0.852152 |
|  |  |  |  |  |  | 0.002157 | 2.16E-07 | 0.000821 | 8.21E-08 | 2.627889 | 0.002009 | 2.01E-07 | 0.002422 | 2.42E-07 | 0.829243 |
| PSialA-3-M | 0.00155 | 1.55E-07 | 0.001559 | 1.56E-07 | 0.994682 | 1.00E-03 | 1.00E-07 | 1.23E-03 | 1.23E-07 | 8.13E-01 | 0.000955 | 9.55E-08 | 0.000481 | 4.81E-08 | 1.985837 |
|  | 0.001394 | 1.39E-07 | 0.001586 | 1.59E-07 | 0.878918 | 7.80E-04 | 7.80E-08 | 7.30E-04 | 7.30E-08 | 1.07E+00 | 0.000903 | 9.03E-08 | 0.000364 | 3.64E-08 | 2.4791 |
|  | 0.001185 | 1.18E-07 | 0.000678 | 6.78E-08 | 1.746703 | 7.00E-04 | 7.00E-08 | 5.85E-04 | 5.85E-08 | 1.20E+00 | 0.000809 | 8.09E-08 | 0.000412 | 4.12E-08 | 1.963812 |
|  | 0.000926 | 9.26E-08 | 0.000692 | 6.92E-08 | 1.337458 | 5.59E-04 | 5.59E-08 | 5.42E-04 | 5.42E-08 | 1.03E+00 | 0.000811 | 8.11E-08 | 0.000381 | 3.81E-08 | 2.129699 |
|  | 0.000855 | 8.55E-08 | 0.000788 | 7.88E-08 | 1.084414 | 5.27E-04 | 5.27E-08 | 4.85E-04 | 4.85E-08 | 1.09E+00 | 0.000521 | 5.21E-08 | 0.000379 | 3.79E-08 | 1.376977 |
|  |  |  |  |  |  |  |  |  |  |  |  |  |  |  |  |
| PSialA-4-M | 1.05E-02 | 1.05E-06 | 5.36E-03 | 5.36E-07 | 1.95E+00 | Image set cannot be processed | | | | | Image set cannot be processed | | | | |
|  | 6.06E-03 | 6.06E-07 | 3.91E-03 | 3.91E-07 | 1.55E+00 |  |  |  |  |  |  |  |  |  |  |
|  | 5.25E-03 | 5.25E-07 | 3.79E-03 | 3.79E-07 | 1.38E+00 |  |  |  |  |  |  |  |  |  |  |
|  | 4.19E-03 | 4.19E-07 | 2.54E-03 | 2.54E-07 | 1.65E+00 |  |  |  |  |  |  |  |  |  |  |
|  | 3.45E-03 | 3.45E-07 | 2.22E-03 | 2.22E-07 | 1.55E+00 |  |  |  |  |  |  |  |  |  |  |
|  |  |  |  |  |  |  |  |  |  |  |  |  |  |  |  |
| FreeHA-1-F | 0.003944 | 3.94E-07 | 0.0038 | 3.8E-07 | 1.037777 | Image set cannot be processed | | | | | Image set cannot be processed | | | | |
|  | 0.001811 | 1.81E-07 | 0.001625 | 1.62E-07 | 1.114468 |  |  |  |  |  |  |  |  |  |  |
|  | 0.001143 | 1.14E-07 | 0.001029 | 1.03E-07 | 1.110941 |  |  |  |  |  |  |  |  |  |  |
|  | 0.000809 | 8.09E-08 | 0.000694 | 6.94E-08 | 1.165416 |  |  |  |  |  |  |  |  |  |  |
|  | 0.000644 | 6.44E-08 | 0.000539 | 5.39E-08 | 1.195866 |  |  |  |  |  |  |  |  |  |  |
|  |  |  |  |  |  |  |  |  |  |  |  |  |  |  |  |
| FreeHA-2-F | Image set cannot be processed | | | | | 0.001584 | 1.58E-07 | 0.002384 | 2.38E-07 | 0.66428 | Image set cannot be processed | | | | |
|  |  |  |  |  |  | 0.001581 | 1.58E-07 | 0.002216 | 2.22E-07 | 0.713553 |  |  |  |  |  |
|  |  |  |  |  |  | 0.001716 | 1.72E-07 | 0.002364 | 2.36E-07 | 0.725982 |  |  |  |  |  |
|  |  |  |  |  |  | 0.001109 | 1.11E-07 | 0.001203 | 1.2E-07 | 0.921772 |  |  |  |  |  |
|  |  |  |  |  |  | 0.001208 | 1.21E-07 | 0.001215 | 1.21E-07 | 0.994239 |  |  |  |  |  |
|  |  |  |  |  |  |  |  |  |  |  |  |  |  |  |  |
| FreeHA-3-M | 0.002119 | 2.12E-07 | 0.001258 | 1.26E-07 | 1.684472 | Image set cannot be processed | | | | | Image set cannot be processed | | | | |
|  | 0.001664 | 1.66E-07 | 0.001225 | 1.22E-07 | 1.35823 |  |  |  |  |  |  |  |  |  |  |
|  | 0.001134 | 1.13E-07 | 0.001024 | 1.02E-07 | 1.107517 |  |  |  |  |  |  |  |  |  |  |
|  | 0.000579 | 5.79E-08 | 0.000737 | 7.37E-08 | 0.7856 |  |  |  |  |  |  |  |  |  |  |
|  | 0.00087 | 8.7E-08 | 0.000942 | 9.42E-08 | 0.92378 |  |  |  |  |  |  |  |  |  |  |
|  |  |  |  |  |  |  |  |  |  |  |  |  |  |  |  |
| FreeHA-4-M | Image set cannot be processed | | | | | 0.001171 | 1.17E-07 | 0.001627 | 1.63E-07 | 0.719765 | 0.003717 | 3.72E-07 | 0.004467 | 4.47E-07 | 0.832029 |
|  |  |  |  |  |  | 0.000873 | 8.73E-08 | 0.001115 | 1.12E-07 | 0.782726 | 0.001884 | 1.88E-07 | 0.002489 | 2.49E-07 | 0.757174 |
|  |  |  |  |  |  | 0.00048 | 4.8E-08 | 0.000554 | 5.54E-08 | 0.866486 | 0.00103 | 1.03E-07 | 0.001347 | 1.35E-07 | 0.764949 |
|  |  |  |  |  |  | 0.000406 | 4.06E-08 | 0.000499 | 4.99E-08 | 0.812656 | 0.000663 | 6.63E-08 | 0.000994 | 9.94E-08 | 0.667262 |
|  |  |  |  |  |  | 0.000298 | 2.98E-08 | 0.000367 | 3.67E-08 | 0.810871 | 0.000671 | 6.71E-08 | 0.00088 | 8.8E-08 | 0.763285 |
|  |  |  |  |  |  |  |  |  |  |  |  |  |  |  |  |
| FreePSialA-1-F | 0.00155 | 1.55E-07 | 0.001559 | 1.56E-07 | 0.994682 | Image set cannot be processed | | | | | 1.67E-03 | 1.67E-07 | 1.94E-03 | 1.94E-07 | 8.57E-01 |
|  | 0.001394 | 1.39E-07 | 0.001586 | 1.59E-07 | 0.878918 |  |  |  |  |  | 1.79E-03 | 1.79E-07 | 1.56E-03 | 1.56E-07 | 1.14E+00 |
|  | 0.001185 | 1.18E-07 | 0.000678 | 6.78E-08 | 1.746703 |  |  |  |  |  | 1.08E-03 | 1.08E-07 | 1.26E-03 | 1.26E-07 | 8.63E-01 |
|  | 0.000926 | 9.26E-08 | 0.000692 | 6.92E-08 | 1.337458 |  |  |  |  |  | 7.41E-04 | 7.41E-08 | 9.88E-04 | 9.88E-08 | 7.50E-01 |
|  | 0.000855 | 8.55E-08 | 0.000788 | 7.88E-08 | 1.084414 |  |  |  |  |  | 3.78E-04 | 3.78E-08 | 9.05E-04 | 9.05E-08 | 4.18E-01 |
|  |  |  |  |  |  |  |  |  |  |  |  |  |  |  |  |
| FreePSialA-2-F | 0.000292 | 2.92E-08 | 0.000222 | 2.22E-08 | 1.315447 | 0.000316 | 3.16E-08 | 0.000267 | 2.67E-08 | 1.182741 | 3.46E-03 | 3.46E-07 | 1.16E-03 | 1.16E-07 | 2.97E+00 |
|  | 0.000693 | 6.93E-08 | 0.000412 | 4.12E-08 | 1.681574 | 0.000168 | 1.68E-08 | 0.000172 | 1.72E-08 | 0.978097 | Issues with Z shift; ignore this time point | | | | |
|  | 0.000449 | 4.49E-08 | 0.000413 | 4.13E-08 | 1.087877 | 5.29E-05 | 5.29E-09 | 6.93E-05 | 6.93E-09 | 0.76293 | 1.33E-04 | 1.33E-08 | 3.36E-04 | 3.36E-08 | 3.95E-01 |
|  | 0.000448 | 4.48E-08 | 0.000359 | 3.59E-08 | 1.245791 | 3.62E-05 | 3.62E-09 | 6.77E-05 | 6.77E-09 | 0.535401 | 3.58E-04 | 3.58E-08 | 2.89E-04 | 2.89E-08 | 1.24E+00 |
|  | 0.000471 | 4.71E-08 | 0.000347 | 3.47E-08 | 1.357633 | 8.15E-06 | 8.15E-10 | 7.2E-05 | 7.2E-09 | 0.113202 | 2.79E-04 | 2.79E-08 | 1.88E-04 | 1.88E-08 | 1.48E+00 |
|  |  |  |  |  |  |  |  |  |  |  |  |  |  |  |  |
| FreePSialA-3-M | Image set cannot be processed | | | | | 0.002138 | 2.14E-07 | 0.002442 | 2.44E-07 | 0.875647 | 5.75E-03 | 5.75E-07 | 2.84E-03 | 2.84E-07 | 2.03E+00 |
|  |  |  |  |  |  | 0.000967 | 9.67E-08 | 0.001499 | 1.5E-07 | 0.644921 | 1.23E-03 | 1.23E-07 | 2.01E-03 | 2.01E-07 | 6.09E-01 |
|  |  |  |  |  |  | 0.001288 | 1.29E-07 | 0.001173 | 1.17E-07 | 1.097507 | Issues with Z shift; ignore these time points | | | | |
|  |  |  |  |  |  | 0.001075 | 1.08E-07 | 0.000846 | 8.46E-08 | 1.270694 |  |  |  |  |  |
|  |  |  |  |  |  | 0.000747 | 7.47E-08 | 0.000351 | 3.51E-08 | 2.130335 | 2.01E-03 | 2.01E-07 | 1.44E-03 | 1.44E-07 | 1.40E+00 |
|  |  |  |  |  |  |  |  |  |  |  |  |  |  |  |  |
| FreePSialA-4-M | 1.98E-03 | 1.98E-07 | 2.73E-03 | 2.73E-07 | 7.26E-01 | Image set cannot be processed | | | | | Image set cannot be processed | | | | |
|  | 1.91E-03 | 1.91E-07 | 1.69E-03 | 1.69E-07 | 1.13E+00 |  |  |  |  |  |  |  |  |  |  |
|  | 3.52E-03 | 3.52E-07 | 2.01E-03 | 2.01E-07 | 1.75E+00 |  |  |  |  |  |  |  |  |  |  |
|  | 2.52E-03 | 2.52E-07 | 1.66E-03 | 1.66E-07 | 1.52E+00 |  |  |  |  |  |  |  |  |  |  |
|  | 2.02E-03 | 2.02E-07 | 1.35E-03 | 1.35E-07 | 1.50E+00 |  |  |  |  |  |  |  |  |  |  |
